# Dysregulation of the Transforming Growth Factor β Pathway in Induced Pluripotent Stem Cells Generated from Patients with Diamond Blackfan Anemia

**DOI:** 10.1371/journal.pone.0134878

**Published:** 2015-08-10

**Authors:** Jingping Ge, Marisa Apicella, Jason A. Mills, Loïc Garçon, Deborah L. French, Mitchell J. Weiss, Monica Bessler, Philip J. Mason

**Affiliations:** 1 Division of Hematology, The Children’s Hospital of Philadelphia, Philadelphia, Pennsylvania, United States of America; 2 Department of Pathology and Laboratory Medicine, The Children’s Hospital of Philadelphia, Philadelphia, Pennsylvania, United States of America; 3 UPMC University Paris 06, UMR_S938, and Assistance Publique- Hôpitaux de Paris, Paris, France; 4 Department of Hematology, St. Jude Children’s Research Hospital, Memphis, Tennessee, United States of America; University of Texas Health Science Ctr, UNITED STATES

## Abstract

Diamond Blackfan Anemia (DBA) is an inherited bone marrow failure syndrome with clinical features of red cell aplasia and variable developmental abnormalities. Most affected patients have heterozygous loss of function mutations in ribosomal protein genes but the pathogenic mechanism is still unknown. We generated induced pluripotent stem cells from DBA patients carrying *RPS19* or *RPL5* mutations. Transcriptome analysis revealed the striking dysregulation of the transforming growth factor β (TGFβ) signaling pathway in DBA lines. Expression of TGFβ target genes, such as *TGFBI*, *BAMBI*, *COL3A1* and *SERPINE1* was significantly increased in the DBA iPSCs. We quantified intermediates in canonical and non-canonical TGFβ pathways and observed a significant increase in the levels of the non-canonical pathway mediator p-JNK in the DBA iPSCs. Moreover, when the mutant cells were corrected by ectopic expression of WT *RPS19* or *RPL5*, levels of p-JNK returned to normal. Surprisingly, nuclear levels of SMAD4, a mediator of canonical TGFβ signaling, were decreased in DBA cells due to increased proteolytic turnover. We also observed the up-regulation of *TGFβ1R*, *TGFβ2*, *CDKN1A* and *SERPINE1* mRNA, and the significant decrease of *GATA1* mRNA in the primitive multilineage progenitors. In summary our observations identify for the first time a dysregulation of the TGFβ pathway in the pathobiology of DBA.

## Introduction

Diamond Blackfan Anemia (DBA) is an inherited bone marrow failure syndrome that presents in early childhood with macrocytic normochromic anemia and sometimes with variable accompanying developmental anomalies, chiefly short stature, thumb abnormalities and cleft palate[1]. In the blood the erythrocyte lineage is the most affected though a stem cell defect, pancytopenia and multilineage bone marrow hypoplasia are present, particularly in older patients[2]. In most patients the disease results from heterozygous mutations or deletions in genes encoding ribosomal proteins (RP), suggesting that haploinsufficiency for RPs triggers the disease[3]. Eleven RP genes encoding proteins of both large and small ribosomal subunits have so far been implicated accounting for about 70% of cases[4]. The mechanism whereby haploinsufficiency for RPs leads to failure of red cell development and other DBA manifestations is unknown. Research into DBA pathogenesis has been hampered by the rarity of the disease and the scarcity of the affected cells, in particular the defective hematopoietic stem cells and precursors. Currently there are two main hypotheses to explain the disease mechanism; one is that RP haploinsufficiency leads to stabilization of p53 and apoptosis of erythroid precursors [5] which require massive ribosome synthesis for globin production though the details of this model have not been rigorously established. Alternatively it is thought that haploinsufficiency of ribosomal proteins may affect translation of specific transcripts required for erythropoiesis[6,7]. In addition some evidence suggests that heme toxicity, which may develop because the rate of globin synthesis lags behind the rate of heme production, may play a role[8].

TGFβ signaling involves a complex network of interacting pathways that regulate many aspects of cellular behavior including control of cellular proliferation, control of extracellular matrix production and degradation, cell migration, invasion and modulation of immune functions[9,10]. The TGFβ superfamily of growth factors exert their effect by binding to Type I or Type II receptors thereby activating receptor kinase activity[11]. In the canonical pathway, signaling to the nucleus occurs via phosphorylation of cytoplasmic SMAD proteins (the name is derived from a *C*.*elegans* protein SMA for small body size and a *Drosophila* protein MAD for mothers against decapentaplegic[12]). Phosphorylated receptor activated SMADs (R-SMADs) bind SMAD4 and are then transported to the nucleus where they act as transcription factors to regulate TGFβ target genes such as those encoding extra-cellular matrix proteins[13]. In addition TGFβ can signal through SMAD-independent non-canonical pathways. These include signal transduction through PI3K/AKT, small GTPases and the MAP kinases ERK or JNK/p38[14]. In hematopoiesis, TGFβ is a potent negative regulator of stem cell proliferation[15,16], and mediates erythroid differentiation [17,18]. TGFβ signaling is dysregulated in many disease phenotypes and is the target of several drugs that have recently been developed, some of which are currently in clinical trials[19,20].

In this paper we show that TGFβ signaling is dysregulated in DBA induced pluripotent stem cells (iPSCs). This identifies a new player in DBA that is implicated in the regulation of the hematopoietic stem cell and in erythroid differentiation and is highly drugable with several compounds already in clinical trial.

## Materials and Methods

### Human iPSC line maintenance and differentiation

The human iPSC lines generated from fibroblasts of DBA patients were described previously[21]. We also used iPSCs generated from mononuclear cells of DBA patients by reprogramming using Sendai virus[22]. The Penn-CHOP Bone Marrow Failure Syndrome (BMFS) cohort is an open prospective/retrospective cohort for the study of molecular mechanisms of BMFS, approved by the Institutional Review Boards of Children’s Hospital of Philadelphia (CHOP) and of the University of Pennsylvania (Penn). Written informed consent from all study participants or their legal guardians was obtained prior to study participation in accordance with the Declaration of Helsinki. “Corrected” lines expressing the WT RP gene were described previously[21]. In many of our experiments we compare multiple clones of wild type, mutant, corrected and mock corrected (expressing GFP) lines. The DBA mutant line is labelled as “*RPS19*/*RPL5* mutant” or “*RPS19*/*RPL5* mutant-”. The mock lines are labelled as “*RPS19*/*RPL5* mutant GFP”. The corrected lines are labelled as “*RPS19/RPL5* mutant *RPS19*/*RPL5*) or “corrected *RPS19*/*RPL5* mutant”. Two wild-type iPSC lines were used in the study. Individual clones of the same type behaved similarly in these experiments. To obtain a homogeneous population of undifferentiated iPSCs, embryoid bodies (EBs) were generated as previously described[21]. iPSCs were differentiated to erthyroid precursor cells (EPCs) as described by Paluru et al. [23]. Three base media were used during the adherent differentiation: RPMI (Invitrogen, Day 0-Day 2), StemPro 34 (Invitrogen, Day 3–5), and serum free differentiation (SFD, Day 7-Day8) medium[24]. Additional cytokines were added throughout the differentiation. The differentiation was maintained at 37°C with 5% CO_2_, 5% O_2_, and 90% N_2_. On Day 8, the derived EPCs (floating cells) were collected and analyzed via FACS Canto II flow cytometer (BD Bioscience, San Jose, CA) for EPCs cell surface markers (S1 Fig).

### Human whole transcript microarray and quantitative Reverse-Transcriptase PCR (qRT-PCR)

Total RNA was isolated from cells using the RNeasy kit (Qiagen, Redwood City, CA). The level of whole genome transcripts was measured by an Affymetrix GeneChip human transcriptome array or human Exon array (Affymetrix, Santa Clara,CA). Data were analyzed by Partek software (Partek, Saint Louis, MO), and pathway analysis carried out using Ingenuity pathway analysis (IPA) (Qiagen) and DAVID Bioinformatics Resources 6.7 (http://david.abcc.ncifcrf.gov/).

cDNA was produced using random hexamers with Superscript III Reverse Transcriptase (Invitrogen). Quantitative PCR reactions were performed using SYBR Green qPCR Master Mix (Roche) and an Applied Biosystems 7500 Fast Real-Time PCR system (Applied Biosystems). The primer sequences are shown S1 Table.

### Immunofluorescence staining

Briefly, iPSCs were cultured on microscope slides, and the slides were then incubated with primary rabbit anti-SMAD4 antibody (Abcam, 1:50). After washes, the slides were incubated in the secondary anti-rabbit Cy3-conjugated antibody (1:500; Jackson ImmunoResearch, West Grove, PA). Slides were also counterstained with DAPI. Slides were viewed under an Axioskop 40 (Zeiss) upright research microscope, and digital images were obtained with a Nikon Coolpix camera.

### Flow cytometry and Western blotting

Antibodies used for flow cytometry are described in S2 Table. Cells were analyzed on a FACSCanto II flow cytometer using FlowJo software (Tree Star Inc.). Protein from whole cell lysate (total protein), nuclear fraction and cytoplasmic fraction was purified using NE-PER nuclear and cytoplasmic extraction reagents (Thermo Fisher). Antibodies for Western blotting included: anti-GAPDH-HRP (Abcam), anti-β actin-HRP (Sigma), anti-fibrillarin (Abcam), anti-SMAD4 (Abcam), anti-SMAD2, 3 (Cell Signaling Tech), anti-JNK (Santa Cruz Biotechonolgy), anti p-JNK (Cell Signaling Tech), anti-p-SMAD2 (Cell Signaling Tech), anti-p-SMAD3 (Millipore), anti-p-SMAD5 (Abcam), anti-USP9x (Abcam), and anti-TIF1γ (TRIM33) (Abcam).

### Statistical analysis

Results from multiple experiments are expressed as the mean ± standard deviation. ANOVA was used in microarray analysis and an unpaired two-tailed Student *t* test was used in q-PCR.

## Results

We have previously produced iPSCs from primary cells from DBA patients[21] heterozygous for the *RPS19* mutation c.376 C>T, p.Q126X, or for the *RPL5* mutation c.67C>T, p.R23X. Both mutations cause haploinsufficiency by introducing an early termination codon. Cell lines heterozygous for mutations in *RPL5* and *RPS19* genes were produced and found to recapitulate crucial biochemical and phenotypic features of the disease. Patient derived fibroblasts were reprogrammed at a very low efficiency with a tendency to spontaneously differentiate. Cell lines showed aberrant ribosome biogenesis and impaired hematopoiesis with erythropoiesis affected the most [21], encouraging us to believe that molecular characterization of these cells may be informative, in an unbiased manner, about the pathogenic mechanisms whereby haploinsufficiency for RPs leads to the abnormal cellular biology and possibly to DBA.

### DBA iPSCs with *RPS19* or *RPL5* mutations show altered TGFβ signaling

In order to investigate the genes and pathways altered by RP haploinsufficiency, we performed an Affymetrix human Exon array comparing the transcriptomes of *RPL5* and *RPS19* mutated DBA iPSCs to wild type iPSCs. Principle component analysis (PCA) showed a good separation of mutant cells from wild type cells (S2 Fig).We identified genes whose expression differed by ≥2-fold between mutant and wild type iPSCs with a probability value < 0.05 and false discovery rate <0.05. By these parameters, expression of 126 genes was increased and that of 89 genes decreased in DBA iPSCs with the *RPL5* mutation; there were 198 genes up-regulated and 70 genes down-regulated in the DBA iPSCs with the *RPS19* mutation. Expression of 72 genes significantly increased in both DBA iPSCs lines, and that of 11 genes significantly decreased in both lines (S3 Table). Differentially expressed genes were analyzed by David and IPA. The most significant pathways identified in both lines were focal adhesion, Extracellular Matrix (ECM) interaction, both of which could be regulated by TGFβ signaling, and TGFβ signaling itself. Genes involved in these pathways are listed in S4 and S5 Tables. Genes involved in focal adhesion and ECM interaction, such as collagens and fibronectin, are also regulated by TGFβ signaling, We selected 11 genes which not only play important roles in TGFβ signaling but were also differentially expressed in both DBA lines relative to control lines (Table 1). Differentially expressed TGFβ related genes that were significantly altered are predicted to regulate cell proliferation, cell differentiation, and extracellular matrix (ECM) production, and include cellular attachment and adhesion proteins.

**Table 1:** Activation of TGFβ signaling in the DBA iPSCs with *RPS19* or *RPL5* mutations.

SYMBOL	GENE NAME	*RPL5* Fold	*RPS19* Fold	p	Comments
TGFBI	Transforming growth factor, β induced	3.705	4.441	0.000	Interacts with ECM
TGFB1	Transforming growth factor, β1	1.666	1.371	0.025	Regulates cell proliferation, differentiation and apoptosis
FN1	Fibronectin 1	3.728	3.677	0.002	Initiation and progression of matrix assembly
SERPINE1	Plasminogen activator inhibitor-1	2.112	2.620	0.001	Classic TGFβ target
ID2	DNA-binding protein inhibitor 2	1.667	2.213	0.010	Negatively regulating cell differentiation
NODAL	Nodal growth differentiation factor	2.420	2.110	0.002	Cell differentiation in early embryogenesis
FST	Follistatin	1.532	2.111	0.006	Binding and bioneutralization of members of the TGFβ superfamily
IGFBP5	Insulin-like growth factor binding protein 5	3.178	5.172	0.001	Interacts with ECM, regulator of cell growth, differentiation or apoptosis
BAMBI	BMP and activin membrane-bound inhibitor	2.556	1.754	0.017	TGFβ negative regulator
SMAD4	SMAD family member 4	0.850	0.695	0.031	Common SMAD in TGFβ pathway to regulate cell proliferation
DKK1	Dickkopf homolog 1	5.381	7.109	0.001	Negative regulator of Wnt signaling

The microarray data was validated by measuring the levels of mRNAs transcribed from specific TGFβ downstream target genes, such as plasminogen activator inhibitor-1 (*PAI-1 or SERPINE1*) and transforming growth factor, β-induced (*TGFBI*) using a second technique, q-PCR (S3 Fig). We observed a significant increase of *SERPINE1* (18.30±1.7 fold for DBA line with *RPS19* mutation; 2.40±0.08 fold for *RPL5* line) and *TGFBI* (8.58±0.34 fold for *RPS19* line; 4.78±0.14 fold for *RPL5* line) in both DBA iPSC lines. Expression of the mRNAs for BMP and activin membrane-bound inhibitor homolog (BAMBI), a transmembrane glycoprotein related to the type I receptors of the TGFβ family, was increased 3.34±0.34 fold in the *RPS19* line and 2.05±0.02 fold in the *RPL5* line. We also observed an increase in the expression of the ECM molecule, collagen type III, alpha 1 gene (*COL3A1*) of 12.97±0.79 for the *RPS19* line and 7.79±0.09 fold for the *RPL5* line. The mRNA levels for these genes were normal in the corrected lines. These results show that TGFβ is significantly altered in iPSC from DBA patients and suggest that altered TGFβ signaling may play a role in DBA pathogenesis.

### Levels of SMAD4, the major effector of the canonical TGFβ signaling pathway are decreased in DBA iPSCs

**Fig 1:**
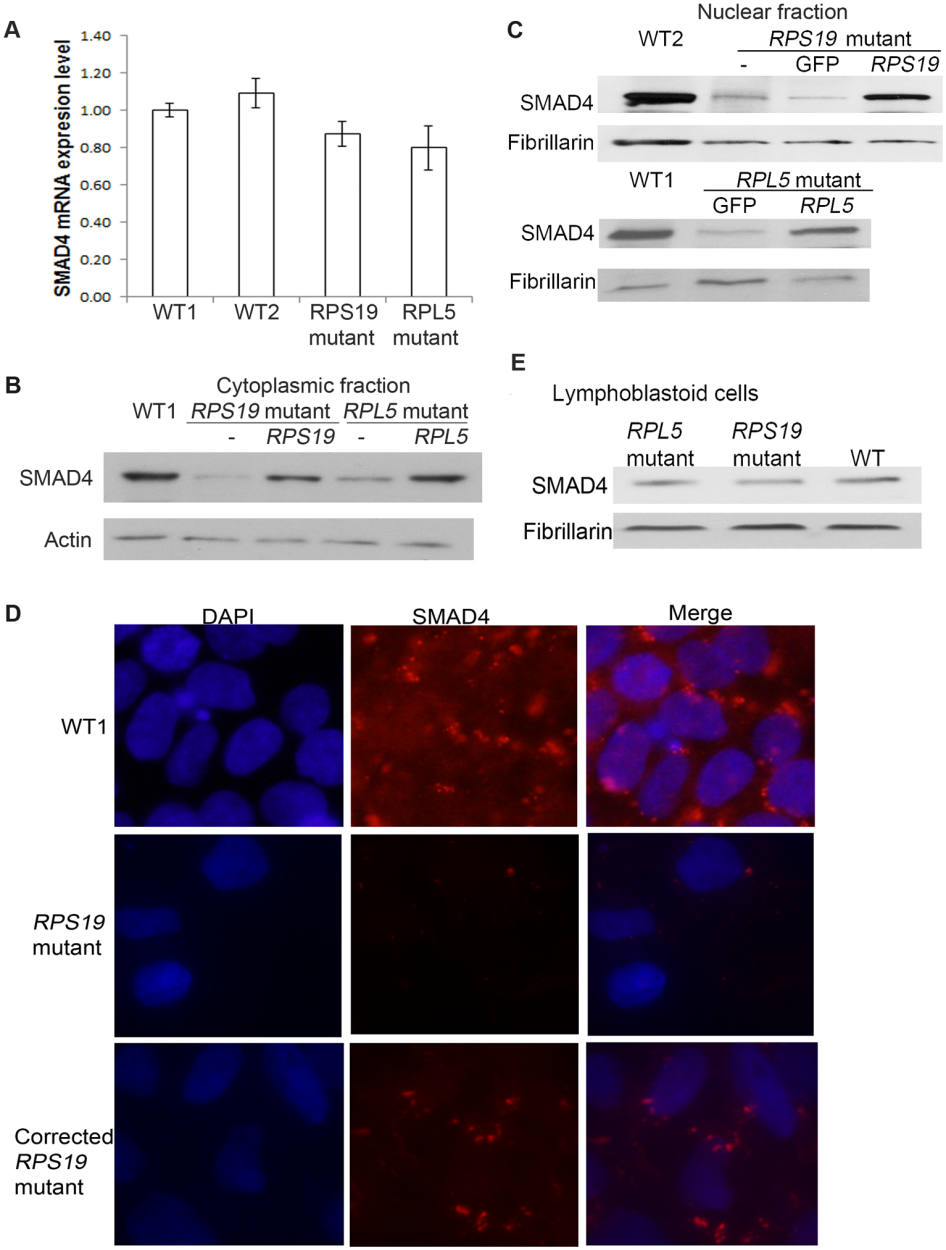
Significant decrease of SMAD4 protein in DBA iPSCs. DBA iPSCs (labelled as “*RPS19/RPL5* mutant” or “*RPS19/RPL5* mutant-”), the corrected DBA cells (expressing a WT version of the mutated gene, *RPL5* or *RPS19*, labelled as “*RPS19/RPL5* mutant *RPS19/RPL5*” or “corrected *RPS19/RPL5* mutant”) and the control GFP cells (expressing GFP, labelled as “*RPS19/RPL5* mutant GFP”) were cultured and the nuclear and cytoplasmic protein was extracted from iPSCs for western blot analysis, and RNA for q-PCR analysis. A) Slight decrease of mRNA level of *SMAD4* in the DBA iPSCs with *RPS19* or *RPL5* mutations. B-C) Dramatic decrease of cytoplasmic and nuclear SMAD4 protein levels in both DBA iPSC lines and SMAD4 levels returned to normal in the corrected lines. D) Immunofluorescence staining showed the decreased SMAD4 levels in DBA iPSCs with *RPS19* mutation compared with those in WT cells, and the corrected line partially restored the SMAD4 protein. Microscope pictures of SMAD4 stained slices were taken using a Leica DM4000B equipped with a 40X objective and image was captured with a Spot RT slider camera. E) No significant decrease of SMAD4 protein in the lymphoblastoid cells generated from the DBA patients, and wild type lymphoblastoid cells was from a healthy control.

The canonical TGFβ pathway is regulated by phosphorylation of the R-SMADs, SMAD2 and SMAD3, followed by the nuclear translocation of the pR-SMAD/SMAD4 complex. Comparing DBA to WT iPSC there was no significant difference in the level of *SMAD4* mRNA (Fig 1A). Since SMAD4 is a transcription factor, the protein level in the nuclear and cytoplasmic fractions was investigated. Western blots revealed a clear decrease in SMAD4 protein levels in the nuclear and cytoplasmic fraction in both DBA lines compared to WT iPSC (Fig 1B and 1C). The decrease of SMAD4 was confirmed by immunofluorescence (Fig 1D). To determine if the decrease in SMAD4 levels was a direct result of the pathogenic mutation, and not an indirect effect of the reprograming procedure, we tested iPSC lines which contained, in addition to the mutated RP gene, a copy of the WT version of the RP gene inserted in the “safe harbor” AAVS1 site. In both *RPL5* and *RPS19* “corrected” lines SMAD4 levels were restored to normal (Fig 1B and 1C). The decrease of SMAD4 protein that we observed in the iPSCs, was not seen in lymphoblastoid cell lines derived from the same DBA patients (Fig 1E). TGFβ signaling via SMAD4 typically inhibits cell proliferation. Reduced SMAD4 protein in DBA iPSC could be a direct result of the haploinsufficency of ribosomal proteins or may represent an adaptive response that confers a proliferative advantage to counter TGFβ pathway mediated growth arrest signals[25].

**Fig 2:**
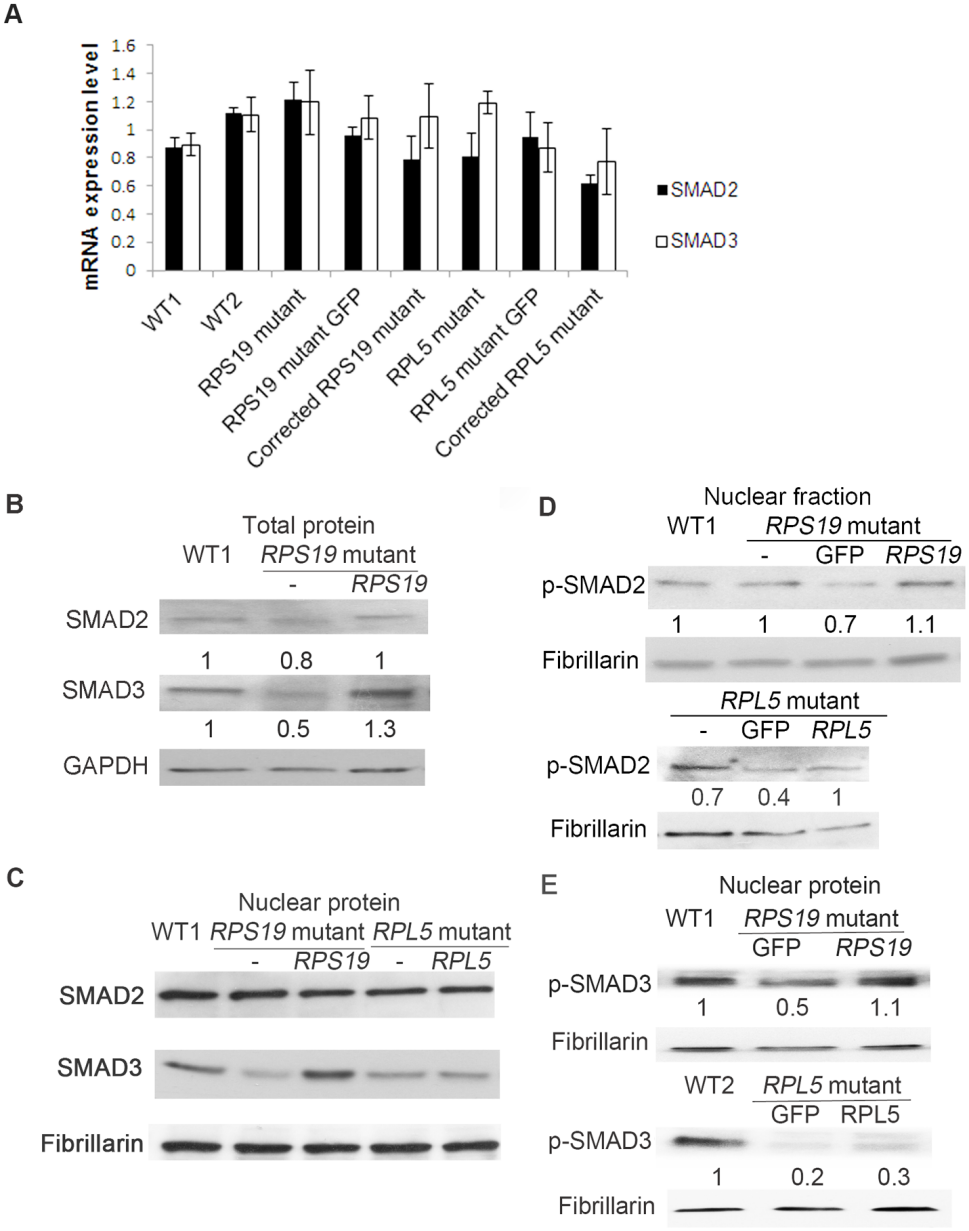
Decrease of SMAD3 protein, but not SMAD2 in DBA iPSCs. DBA iPSCs, the corrected DBA cells, and the control GFP cells were cultured, and the RNA, total protein, protein from nuclear fraction were extracted from iPSCs. The ratio of protein level to WT is labelled under the image. A) No change of mRNA level of *SMAD2* or *SMAD3* in the DBA iPSCs with *RPS19* or *RPL5* mutations. B) Western blot analysis of total protein showing some decrease in SMAD3 protein but the change of SMAD2 was not obvious. C) Western blot analysis of nuclear protein showing decrease in SMAD3 protein in both DBA lines, and no change in SMAD2 protein. D) Western blot analysis showing slight change of p-SMAD2 level in the nuclear fractions from *RPS19* mutant compared to WT and the corrected lines. The nuclear p-SMAD2 decreased in *RPL5* mutant and corrected lines. E) Western blot showing the decrease of p-SMAD3 in the nuclear fraction in both DBA iPS lines, and the nuclear p-SMAD3 protein partially restored in the corrected DBA iPSCs in the *RPS19* corrected line, but not in *RPL5* corrected line.

Since the SMAD4 molecule forms a complex with R-SMADs, and the complex acts as a transcription factor, the levels of SMAD2, SMAD3 and their active forms, p-RSMADs, especially in the nuclear fraction, were investigated. The relative expression of SMAD2 and SMAD3 mRNA were not changed (Fig 2A). The protein level of total and nuclear SMAD3 decreased, and the SMAD2 level was slightly affected (Fig 2B and 2C). The active p-SMAD was measured in the nuclear fraction, since p-SMAD2, p-SMAD3 and SMAD4 enter the nucleus and act as a transcription factor complex together. The levels of p-SMAD2 were not altered in nuclear fractions of the DBA iPSCs with an *RPS19* mutation (Fig 2D). However, the level of p-SMAD3 decreased in the nuclear fraction of the *RPS19* mutant line, and p-SMAD3 remained at a relatively normal level in the corrected lines (Fig 2E). In iPSCs with an *RPL5* mutation, the p-SMAD2 and p-SMAD3 both decreased in the mutant line. In the canonical TGFβ pathway, SMAD2 and SMAD3 play equal roles as R-SMADs, and later bind to SMAD4 prior to the complex translocating into the nucleus. However, SMAD3 also plays a role in the non-canonical pathway[26]. In the *RPS19* mutant lines, we only observed decreased SMAD3, but not SMAD2, indicating that the change of SMAD3 and SMAD4 was not regulated solely by canonical TGFβ signaling. SMAD1, SMAD5, and SMAD8 can also interact with SMAD4 to form the complex through the BMP pathway. We did not observe any changes in *SMAD5* mRNA or nuclear p-SMAD5 in the *RPS19* mutant lines, which indicated that the change of SMAD4 was not affected by the BMP pathway (S4A and S4B Fig).

### Mild effect on SMAD4 in DBA iPSCs after treatment of TGFβ1 activator and TGFβ inhibitor

**Fig 3:**
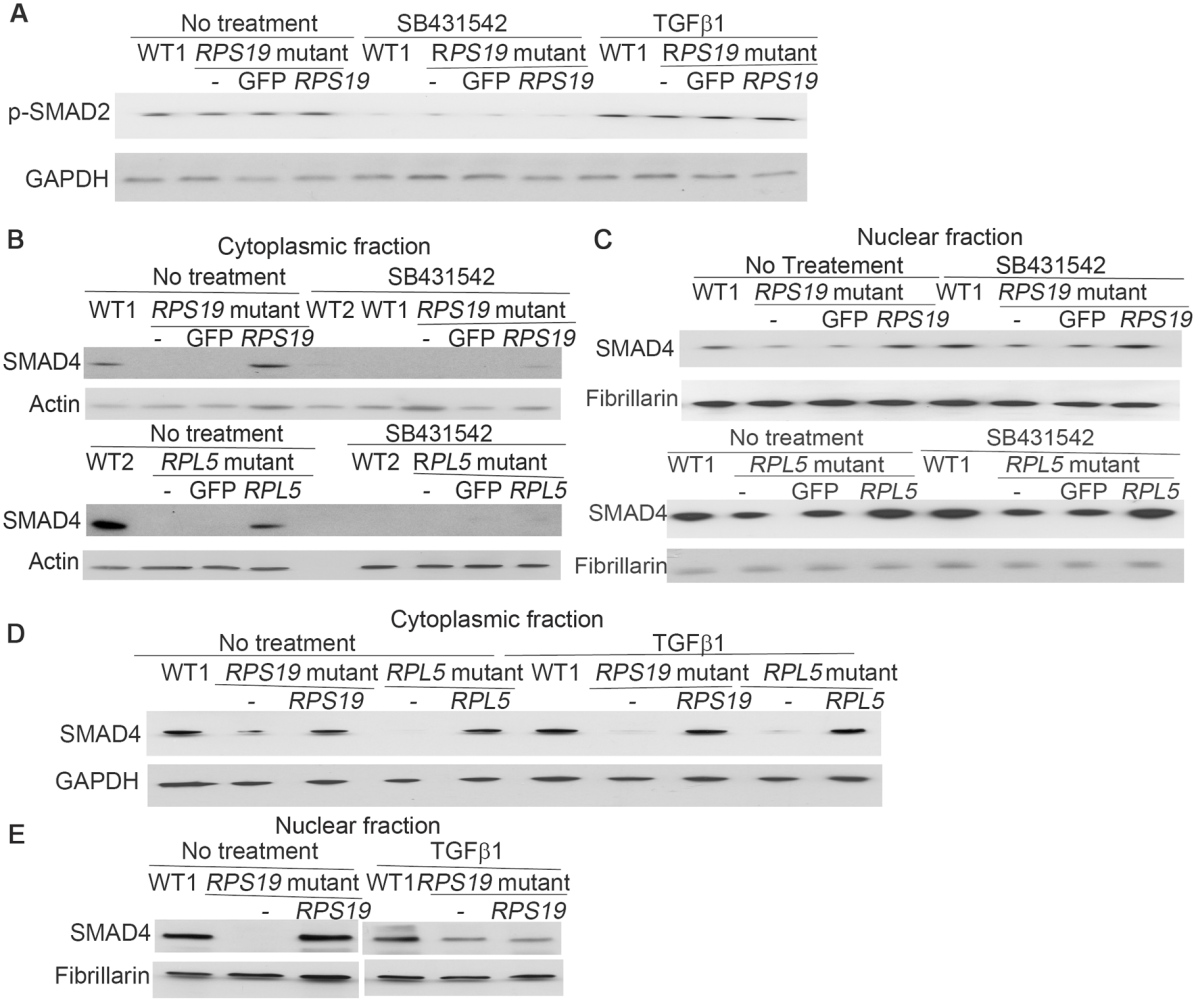
Change of SMAD4 after TGFβ inhibitor /activator treatment. The iPSCs were cultured in iPSC medium, and treated with TGFβ inhibitor SB431542 (10μM) for 3 days or treated with TGFβ activator, TGFβ1 (20ng/ml) for 24h. DMSO treatment was used as the control. Protein levels were measured by Western blot analysis. A) Decrease of total p-SMAD2 after SB431542 treatment in all iPSCs, and increase of p-SMAD2 in both DBA iPSCs after the TGFβ1 treatment. B) Decrease of SMAD4 in the cytoplasmic fraction of all iPSCs after SB431542 treatment. C) Slight accumulation of SMAD4 in the nucleus after SB431542 treatment in DBA iPSCs with *RPS19* or *RPL5* mutations. D-E) Mild increase of cytoplasmic and nuclear SMAD4 in DBA iPSCs after the TGFβ1 treatment.

IPA analysis suggested that the TGFβ pathway was activated in DBA iPSC. Since SMAD4 is the common nuclear effector of TGFβ signaling we reasoned that, if TGFβ signaling was active TGFβ inhibitors would decrease nuclear SMAD4 levels and activators would increase the levels. SB431542 is a potent and specific inhibitor of TGFβ type I activin receptor-like kinase (ALK) receptors. Treatment with SB431542 reduced the level of p-SMAD2 in DBA and WT iPSCs (Fig 3A), indicating the inhibition of TGFβ signaling in these treated cells. The treatment with TGFβ1, on the other hand, increased the protein level of p-SMAD2 (Fig 3A), indicating the activation of TGFβ signaling in the cells. Treatment with SB431542 decreased the protein level of cytoplasmic SMAD4, and slightly increased it in the nuclear fraction (Fig 3B and 3C), while treatment with TGFβ1 slightly increased the cytoplasmic and nuclear SMAD4 in the DBA iPSCs (Fig 3D and 3E). Nuclear SMAD4 levels are consistently higher in cells corrected by expression of the deficient RP than cells with haploinsufficiency. These results suggest that TGFβ signaling is operating to facilitate phosphorylation of-SMAD2, and TGFβ inhibitor inhibits the SMAD2 phosphorylation, which blocks the nuclear translocation. However, the level of nuclear SMAD4 is not regulated only or directly by TGFβ, and it may be affected by multiple factors in the complex control of cell growth. Therefore, the decrease of SMAD4 that we observe in the DBA iPSCs is not directly caused by TGFβ signaling.

### Activation of p-JNK in the non-canonical TGFβ pathway

**Fig 4:**
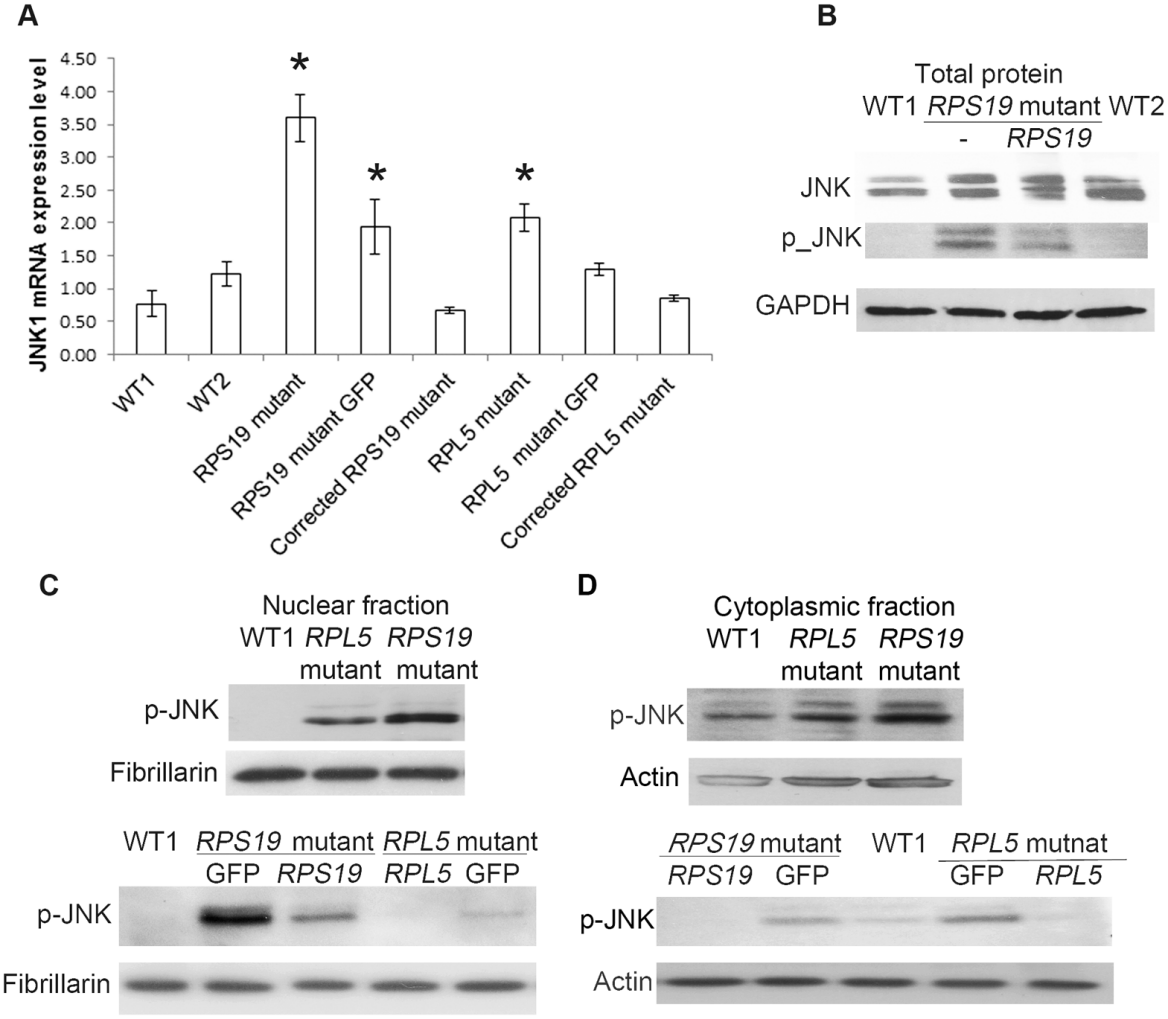
Activation of non-canonical TGFβ pathway through p-JNK. DBA iPSCs, the corrected DBA cells and the GFP cells were cultured, and RNA, total protein and the protein from nuclear and cytoplasmic fraction were extracted from iPSCs for analysis. Fibrillarin were used as loading controls. A) Overexpression of *JNK1* mRNA level in both DBA mutant lines. B) Elevation of JNK protein, especially p-JNK, in the total protein of DBA iPSCs with *RPS19* mutation. C) Accumulation of nuclear p-JNK in the DBA iPSCs and the GFP lines (especially in *RPS19* mutant line), and restoration of p-JNK levels in the corrected DBA lines. D) Increase of cytoplasmic p-JNK in the DBA iPSCs, and normal levels of p-JNK in the rescued DBA lines.

As well as its role in canonical TGFβ signaling SMAD3 can also be involved in the non-canonical pathway. In non-canonical TGFβ pathways different signaling pathways respond to activation of TGFβ receptors and transduce the signal to the nucleus via a variety of molecules, that may be part of other signaling pathways, to regulate a wide and complex variety of cellular responses. Three such pathways act through the p38, ERK and JNK map kinase cascades. To determine if these non-canonical pathways were operating in the DBA iPSC we measured the levels of p-ERK, p-p38 and p-JNK in the DBA cells and compared these with the levels in iPSC from healthy controls. We measured the protein level of p-ERK, and did not observe any difference between DBA and control iPSCs (S5 Fig). We did not detect the presence of p-p38 in the DBA derived lines. However, we observed a change in the JNK molecule. The relative expression of *JNK1* mRNA increased in both mutant lines (Fig 4A). The JNK protein level increased and we observed an increase of p-JNK in the *RPS19* mutant line (Fig 4B). The p-JNK levels were elevated in the nuclear and cytoplasmic fractions of both lines of DBA iPSCs, with a greater increase in the DBA iPSCs with an *RPS19* mutation (Fig 4C and 4D). The amount of p-JNK in the corrected lines was only slightly increased compared to the amount in the wild-type cells, and was very much decreased compared with the DBA cells.

Cross-talk between JNK and TGFβ is known, and JNK signaling may have either a positive or a negative effect on TGFβ signaling[27,28]. In our DBA iPSCs, we observed a decrease of SMAD3 and p-SMAD3 (Fig 2B). The activation of non-canonical TGFβ has been reported to activate PAI-1 and collagens[29,30], and the activation of p-JNK molecules can lead to the inactivation of SMAD3 as a regulatory mechanism to maintain cell growth[26]. This may also account for the decrease in SMAD4 in the nucleus.

### Rapid turnover of SMAD4

**Fig 5:**
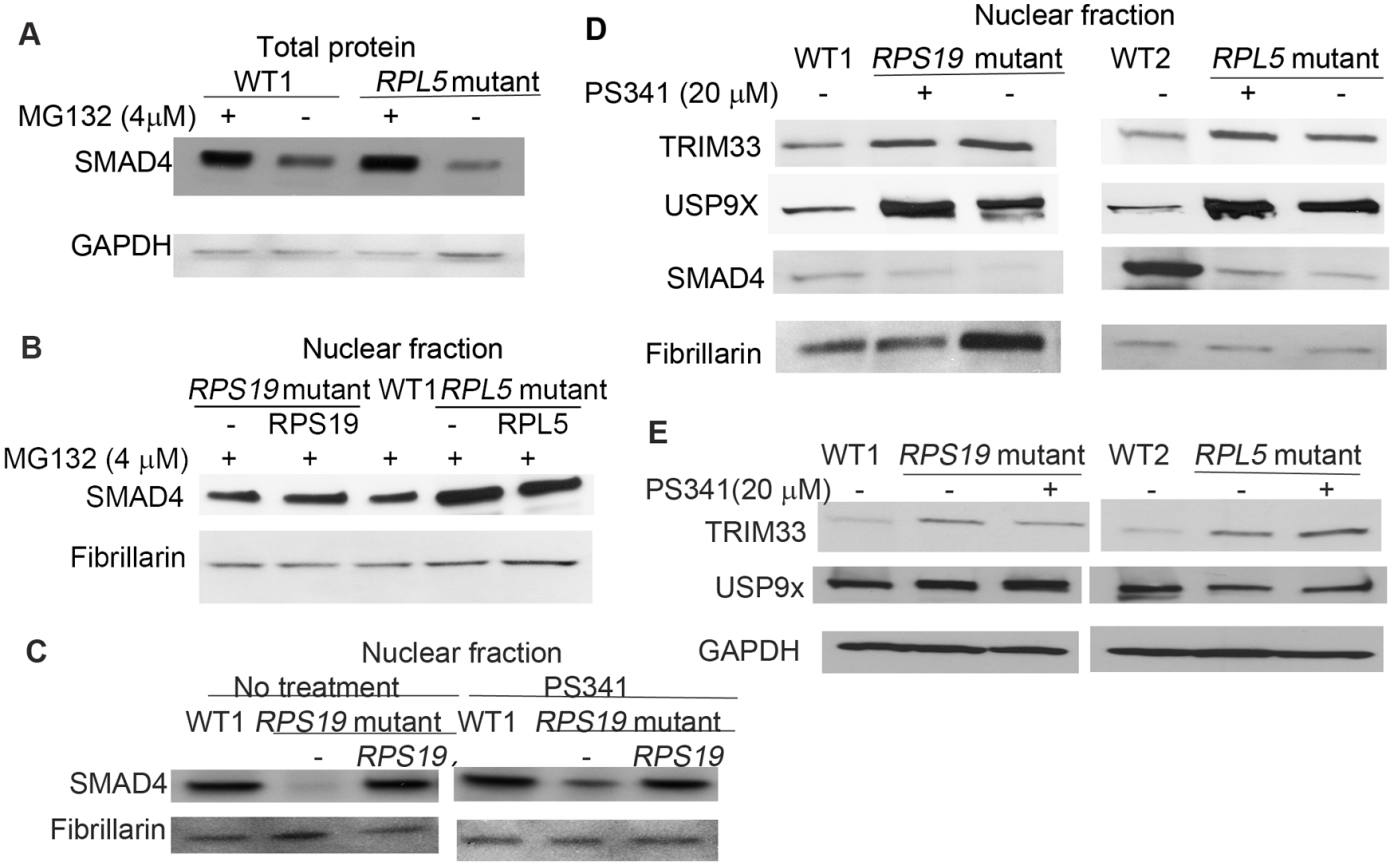
Rapid proteasome degradation of SMAD4 in DBA iPSCs. DBA iPSCs, and the corrected DBA cells were cultured in the iPSC medium, and treated with proteasome inhibitors MG132 (4 μM) for 12h or PS341 (20 μM) for 4h. A-B) Increased level of SMAD4 in both DBA cells after treatment with proteasome inhibitor MG132 for 12h. C) Increased level of nuclear SMAD4 in DBA iPSCs with *RPS19* mutation after PS341 treatment. D and E) Increase of nuclear and cytoplasmic TRIM33, and increase of nuclear USP9X in both DBA iPSCs. The PS341 treatment did not affect the levels of TRIM33 and USP9X.

Although we observed decreased levels of SMAD4 in the DBA iPSC there was little change in *SMAD4* mRNA levels, suggesting either reduced translation or increased protein degradation. Treatment with protein synthesis inhibitor cycloheximide decreased the SMAD4 protein level in DBA and WT iPSCs, but did not change the ratio of SMAD4 protein between DBA and WT cells (S6 Fig). This implies that turnover of SMAD4 is altered in DBA iPSC. Next, we investigated the degradation of SMAD4. The level of SMAD4 was measured after treatment with proteasome inhibitor MG132[31], and we observed the accumulation of SMAD4, especially in nuclear fraction, in both DBA iPSC lines, confirming that SMAD4 is turned over in a proteasome dependent manner (Fig 5A and 5B and S6 Fig). Treatment with PS341[32], a proteasome inhibitor clinically used to treat multiple myeloma, showed some accumulation of nuclear SMAD4 (Fig 5C and 5D). Therefore, we conclude that rapid turnover of SMAD4 in the DBA iPSCs proceeds via ubiquitin-proteasome degradation.

Many TGFβ pathway protein components are regulated by ubiquitin dependent proteolysis at the proteasome[33]. TRIM33 (TIF1γ) serves as a SMAD4 monoubiquitin ligase, and monoubiquitination of SMAD4 disrupts the SMAD2/3-SMAD4 complex, which leads to SMAD4 translocation to the cytosol [34]. USP9x is a deubiquitinating enzyme that acts on ubiquitinated SMAD4[35]. Together these proteins regulate SMAD4 ubiquitination, a crucial step in TGFβ signaling. We observe the levels of nuclear USP9x and TRIM33 are increased in the DBA iPSCs compared to the wild-type (Fig 5D and 5E). This observation suggests that the regulation of SMAD4 ubiquitination is altered in DBA iPSCs though the exact mechanism remains obscure.

### Activation of the TGFβ pathway in differentiated hematopoietic cells

**Fig 6:**
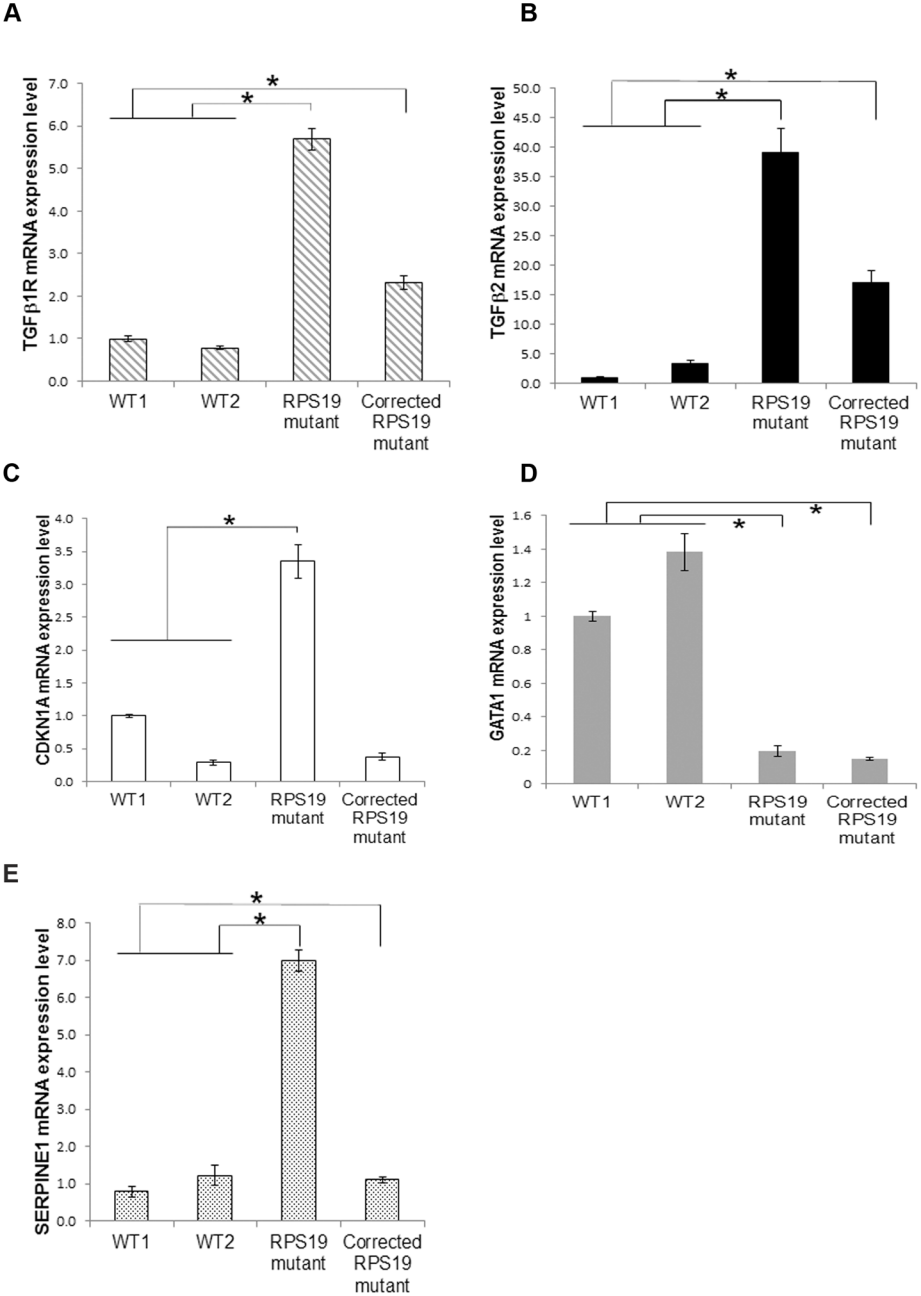
Activation of the TGFβ pathway, and down-regulation of *GATA1* in the differentiated DBA hematopoietic cells. The DBA iPSCs underwent the erythroid differentiation protocol for 8 days. The CD41+ CD235+ multilineage pluoripotent progenitor cells were collected, and RNA was purified for q-PCR. A) Up-regulation of *TGFβ1R* mRNA level. B) Up-regulation of *TGFβ2* mRNA. C) Up-regulation of *CDKN1A* mRNA level. D) Down-regulation of the mRNA of *GATA1*. E) Up-regulation of *SERPINE1* mRNA level. * p<0.05

We have shown that non-canonical TGFβ signaling is activated in iPSCs from DBA patients which may partially explain the hematopoietic phenotype of DBA since TGFβ signaling is inhibitory in hematopoiesis. We previously showed that the DBA iPSC were deficient in erythrocyte production in an *in vitro* differentiation assay[21]. We were therefore interested in the TGFβ signaling in differentiating cells. DBA iPSC were differentiated along the erythrocyte lineage by sequential cytokine treatment. We previously reported a dramatic decrease not only in total hematopoietic cells but also in erythroid cells from day 9 during differentiation[21]. In order to identify genes whose expression may be relevant to the process of erythroid differentiation, we collected the floating cells, which contain the CD41^+^CD235^+^ primitive multilineage progenitor population, at day 8. We studied the gene expression using Affymetrics Genechip Human transcriptome microarray and real-time quantitative PCR. By comparing the transcriptomes of the DBA iPSCs with an *RPS19* mutation with the corrected lines and wild-type lines, we analyzed genes significantly changed in the DBA cells (≥2 fold different; p<0.05, FDR<0.05). Among these genes, 48 genes were restored in the *RPS19* corrected lines (S6 Table). PCA showed good distinction between the DBA and wild type cells, which indicates different expression patterns between hematopoietic cells in DBA and WT cells (S7 Fig). It also showed the separation of DBA cells (*RPS19* mutation) from the corrected *RPS19* cells, again showing distinct gene expression patterns between DBA cells and corrected cells. With IPA an unbiased survey of the DBA hematopoietic gene dataset revealed the significantly changed pathways and molecular and cellular functions in DBA cells were cell growth, cell death, and cell cycle related (S7 Table). The top upstream regulator of these pathways was TGFβ1 (S8 Table), which was predicted to be activated in the DBA cells at day 8. We observed genes related to cell cycle regulation and G1 arrest, such as *CDKN1A (p21)*, *TP53*, *TGFB1R*, *TGFβ2*, and *GATA1*. q-PCR analysis confirmed the up-regulation of *TGFβ1R*, *TGFβ2*, *SERPINE1* and *CDKN1A* in the primitive multilineage progenitors and the significant decrease of *GATA1* mRNA in the progenitor cells (Fig 6A–6E).

## Discussion

**Fig 7:**
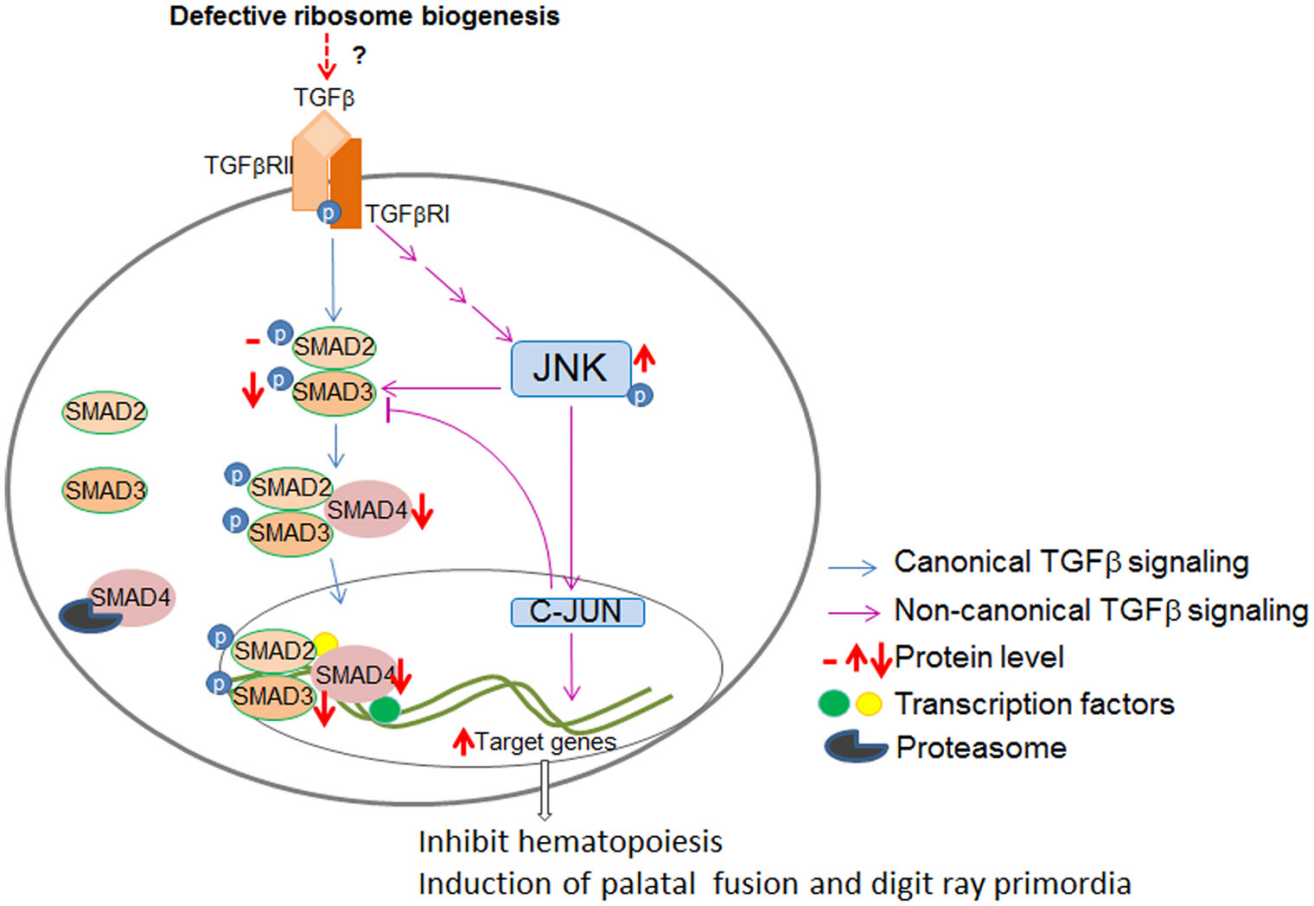
Molecular model of dysregulation of TGFβ signaling in DBA iPSCs. Ribosomal haploinsufficiency may lead to an increasing expression of TGFβ downstream targets through non-canonical JNK signaling. We also identified an increased proteolytic turnover of SMAD4 in the DBA iPSCs. The activation of TGFβ signaling contributes to the reduced differentiation of erythroid cells, and the decrease of SMAD4 could be the major factor permitting DBA iPSCs to proliferate.

Based on the results we have presented here, we observed that the TGFβ signaling is dysregulated in the DBA iPSCs, and we hypothesize that RP haploinsufficiency leads to an increasing expression of downstream TGFβ targets through non-canonical signaling (Fig 7). We also identified an increased proteolytic turnover of SMAD4 in the DBA iPSCs, and we hypothesize the decrease of SMAD4 is the major factor permitting DBA iPSCs to proliferate[26]. We initially described the difficulties of obtaining DBA iPSCs and their tendency to differentiate [21]. Increased TGFβ signaling would at least in part explain this phenotype[36]. Only a few clones were isolated that were able to be maintained as undifferentiated iPSCs, possibly due to activation of a pathway that decreases the TGFβ downstream effect. Thus the activation of the TGFβ pathway may cause the anti-proliferative effect in DBA whereas the downregulation of SMAD4 may permit the cells to divide and grow. This mechanism is supported by our observation that production of DBA iPSCs is inefficient compared with other iPSCs. These results, if they accurately reflect hematopoiesis in DBA patients, suggest that therapeutic manipulation of TGFβ signaling may be beneficial in DBA.

In the majority of DBA patients the disease is caused by heterozygous mutations in one of a subset of ribosomal protein genes. Inheritance is therefore either dominant, or sporadic due to *de novo* mutations. We investigated iPSCs with *RPS19* or *RPL5* mutations, and our study focused on the molecules and pathways that were similar in both mutant cell lines. This strategy strengthens our findings to explain the molecular mechanism of DBA, although we did observe some differences between the two mutations, such as the p-SMAD2 level. In fact there are phenotypic differences between patients with *RPL5* and *RPS19* mutations, notably an increase in somatic abnormalities in patients with a *RPL5* mutation[37].

Recently several patients diagnosed with DBA have been found to have mutations in the X-linked *GATA1* gene which encodes the erythroid/megakaryocyte transcription factor GATA-1[7,38–40]. 20–30% of DBA cases are due to as yet undetermined causes[41]. The mechanism whereby haploinsufficiency for ribosomal proteins, which would be predicted to affect all cells, causes the specific phenotypic effects seen in DBA, is not understood. We observed an increase in *TGFβ2*, *TGFβ1R*, *CDKN1A* and *SERPINE1*, and a decrease in *GATA1* mRNA in the DBA hematopoietic cells at day 8 after differentiation. *RPS19* deficiency leads to inflammation, p53 activation, and decreased *GATA1* expression in the hematopoietic stem/progenitor cells[42]. Inhibition of TGFβ1 signaling normalizes the expression of p53-related genes, restoring hematopoiesis[43]. Our results suggest that the ribosomal haploinsufficiency can lead to activation of TGFβ signaling, activation of the p53 pathway, and the decrease of *GATA1* expression during hematopoiesis. These results may partially explain the finding that some patients diagnosed with DBA have mutations in *GATA1* and strengthen the identification of *GATA1* as a DBA gene.

As well as erythroid deficiency the defects in DBA, which vary in penetrance, include short stature, cleft palate, developmental abnormalities affecting the digits, particularly the thumbs, and cardiovascular defects. The most popular theory is that haploinsufficiency for a ribosomal protein may disturb ribosome biogenesis in cells like erythroid precursors which are very active in ribosome production. This may lead to an excess of some RPs including those, like RPL11 and RPL5, that can bind to and inhibit the E3 ubiquitin ligase MDM2[44–46]. Since MDM2 ubiquitinates and accelerates the degradation of p53 this can lead to increased p53 levels, cell cycle arrest and apoptosis, which may explain the defective red cell production and possibly other DBA features. Our finding of activation of TGFβ signaling adds another factor to the unfolding story. Although we do not know the mechanism via which RP haploinsufficiency gives rise to elevated TGFβ signaling our finding that correcting DBA cells by expressing more of the RP that is lacking suggests a direct connection. It is interesting to note that increased TGFβ signaling is known to inhibit hematopoiesis at the stem cell level and controls red cell differentiation. Furthermore the TGFβ-SMAD4 pathway plays a crucial developmental role in palatal fusion[47], and the induction of digit ray primordia[48]. Thus disturbances in TGFβ signaling could explain the co-expression of the hematopoietic and developmental defects in DBA.

Curiously the levels of the major nuclear effector of canonical TGFβ signaling, SMAD4, are actually decreased in the DBA iPSCs. SMAD4 mediates growth inhibition, and the absence of endothelial SMAD4 causes an increased expression of bone morphogenetic protein 4 which leads to the excessive generation of blood cells[49]. The decreased levels of SMAD4 in DBA cells may have arisen due to a growth advantage during cell proliferation, whereby DBA cells with lower SMAD4 protein outgrow those with higher levels. One of the proteins that regulate SMAD4 is the E3 ubiquitin-protein ligase TRIM33, which promotes SMAD4 ubiquitination, nuclear exclusion and degradation via the ubiquitin proteasome pathway. We observed an increased level of TRIM33, which may explain the decreased SMAD4 level in the DBA iPSCs due to rapid degradation. TRIM33 was also reported to act as a transcription elongation factor in erythroid cells and mediate erythroid differentiation in response to TGF β[50]. In human hematopoietic progenitor cells, TRIM33 and SMAD4 competitively bind to SMAD2/3. Therefore, the TRIM33-SMAD2/3 and SMAD4-SMAD2/3 complex function as complementary effectors to control hematopoietic cell fate by the TGFβ/SMAD[50].

The involvement of TGFβ signaling in many cellular processes and its dysregulated expression in many disease states, including cancer, cardiovascular disease and fibrosis have led to the development of multiple drugs that target different components of TGFβ signaling pathways. A more detailed understanding of the aberration in the TGFβ pathway in DBA, and how it affects hematopoiesis and red cell differentiation might identify a suitable target to improve blood cell production at the level of the hematopoietic stem cell and in erythroid differentiation in patients with DBA. Interestingly a drug, sotatercept, an activin receptor type IIA ligand trap, acts by inhibiting signaling downstream of activin and other TGFβ superfamily members is currently being tested in patients with DBA (ClinicalTrials.gov https://clinicaltrials.gov/ct2/show/NCT01464164) [51]. Sotatercept was originally designed as a treatment to improve bone mineral density in menopausal women[20]. In early clinical trials it was found that women treated with this drug rapidly developed increased numbers of red blood cells and increased hemoglobin, leading to the establishment of clinical trials for the drug in β-thalassemia, MDS and DBA[52]. Whether sotatercept interferes with aberrant TGFβ signaling at the appropriate stage of red cell differentiation and thus will sustainably rescue red cell differentiation or hematopoiesis in DBA iPSCs and in patients with DBA remains to be determined.

## Supporting Information

S1 Fig Differentiated DBA cells with *RPS19* mutation at day 8. iPSCs were differentiated to erythyroid precursor cells (EPCs) as described by Paluru et al. 25. On Day 8, the derived EPCs was collected and analyzed via FACS for EPCs cell surface markers. We observed a significantly decrease in the CD41+ CD235+ primitive multilineage progenitor population.
(TIF)

S2 Fig Principle component analysis (PCA) showed a good separation of DBA mutant iPSCs from wild type cells. RNA from iPSCs was used for Affymetrix Genechip human exon microarray, and PCA was performed to show overall gene expression difference between DBA mutant iPSCs and wild type iPSCs.
(TIF)

S3 Fig TGFβ downstream genes are upregulated in DBA iPSCs with *RPS19* or *RPL5* mutations. DBA iPSCs were cultured in iPSC medium for 2 days to obtain a homogeneous population of undifferentiated iPSCs, and RNA was extracted for q-PCR. The TGFβ down-stream genes, such as *PAI-1*, *COL3A1*, *TGFBI*, and *BAMBI*, were measured in DBA iPSCs with *RPS19* or *RLP5* mutations, and we observed a significant increase in the DBA iPSCs compared to the levels in the wild-type cells. The corrected lines showed a drastic decrease compared to mutant lines. *p<0.05 compared to wildtype.
(TIF)

S4 Fig Expression of SMAD5 in both DBA iPS lines. DBA iPSCs were cultured in iPSC medium for 2 days. RNA and protein was extracted for q-PCR and western blot. A) No change of *SMAD5* mRNA level in DBA iPSCs with *RPS19* or *RPL5* mutations. B) Western blot showed no significant change of nuclear p-SMAD5 level in two DBA lines with the same *RPS19* mutation from different patients.
(TIF)

S5 Fig No change of p-Erk protein level in both DBA mutant lines as well as corrected lines. DBA iPSCs were cultured in iPSC medium for 2 days, and protein was extracted for western blot. We observed no change of p-Erk protein in DBA cells compared to wild type cells.
(TIF)

S6 Fig Level of SMAD4 after cycloheximide treatment in both DBA mutant lines. DBA iPSCs were cultured in iPSC medium for 2 days, and protein was extracted for western blot. Treatment with protein synthesis inhibitor cycloheximide decreased the SMAD4 protein level in DBA and WT iPSCs, but did not change the ratio of SMAD4 protein between DBA and wild type cells. The gel also shows the effect of MG132 on SMAD4 levels in the *RPS19* mutant cell line which are also the subject of Fig 5B.
(TIF)

S7 Fig PCA showed a good separation of multilineage progenitors with an *RPS19* mutant from wild type cells and corrected line. iPSCs were differentiated to EPCs as described by Paluru et al. 25., RNA from derived multilineage progenitors on Day 8 was used for Affymetrix Genechip human transcriptome microarray. PCA was performed to show overall gene expression difference among DBA cells, corrected cells and wild type cells.
(TIF)

S1 Table Primers used for q-PCR. (DOCX)

S2 Table Antibodies that were used in the flow cytometry. (DOCX)

S3 Table Common genes whose expression differed by 2-fold or more between DBA iPSCs with *RPS19* or *RPL5* mutations and wild type iPSCs. (DOCX)

S4 Table David pathway analysis of iPSCs with *RPS19* mutation. (DOCX)

S5 Table David pathway analysis summary of iPSCs with *RPL5* mutation. (DOCX)

S6 Table Genes were significantly changed in DBA cells with *RPS19* mutation compared with the wild-type and restored in the correct lines at day 8 differentiation. (DOCX)

S7 Table Ingenuity analysis of pathways and molecular and cellular functions in hematopoietic progenitors with *RPS19* mutation on day 8. (DOCX)

S8 Table Ingenuity analysis of upstream regulators in hematopoietic progenitors with *RPS19* mutation on day 8. (DOCX)
