# Supplementary figures and images for: Dysregulation of the Transforming Growth Factor β Pathway in Induced Pluripotent Stem Cells Generated from Patients with Diamond Blackfan Anemia
Source: PLoS One. 2015 Aug 10;10(8):e0134878. doi: 10.1371/journal.pone.0134878 (PMC4530889; doi:10.1371/journal.pone.0134878)

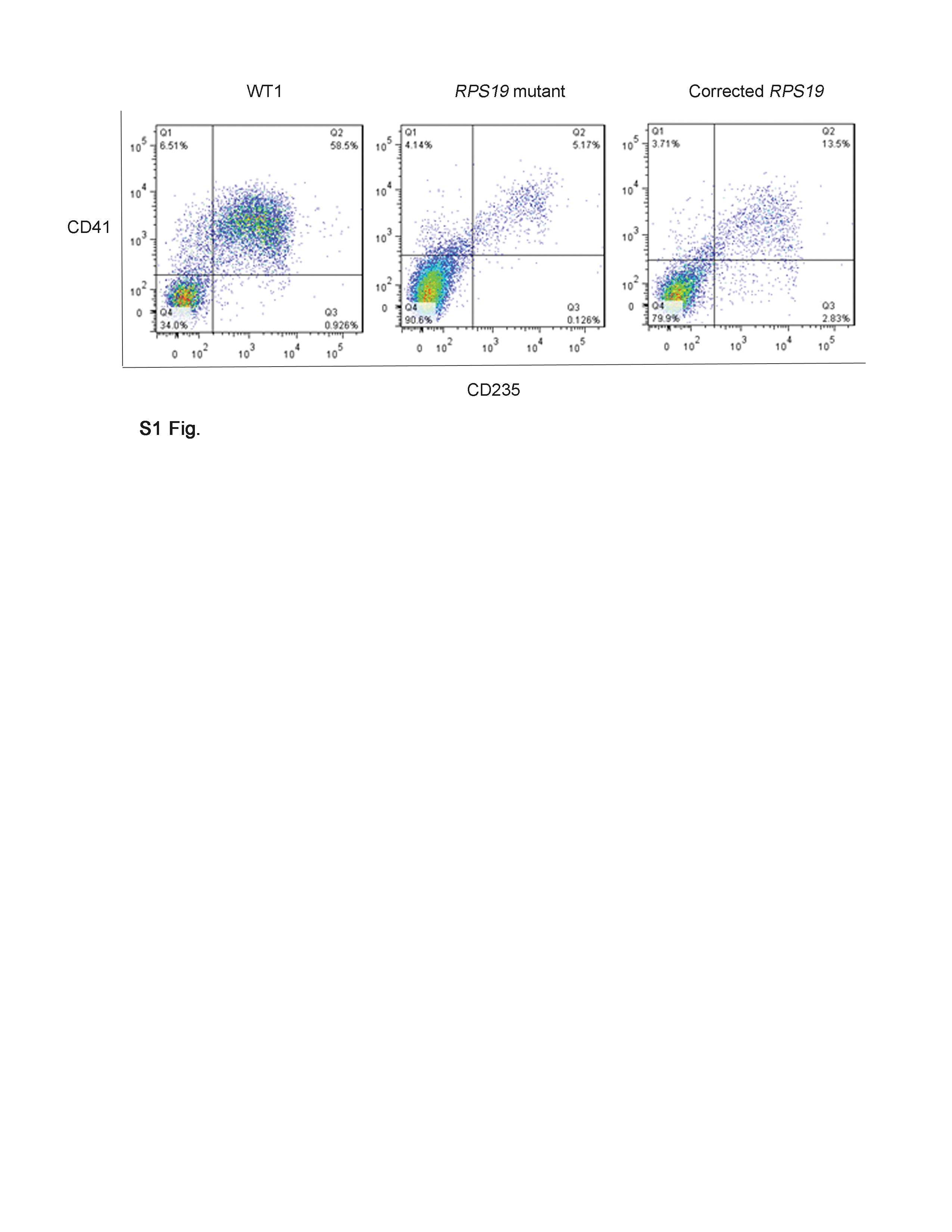

Supplement: S1 Fig — iPSCs were differentiated to erythyroid precursor cells (EPCs) as described by Paluru et al. 25. On Day 8, the derived EPCs was collected and analyzed via FACS for EPCs cell surface markers. We observed a significantly decrease in the CD41+ CD235+ primitive multilineage progenitor population. (TIF) [file pone.0134878.s001.tif]

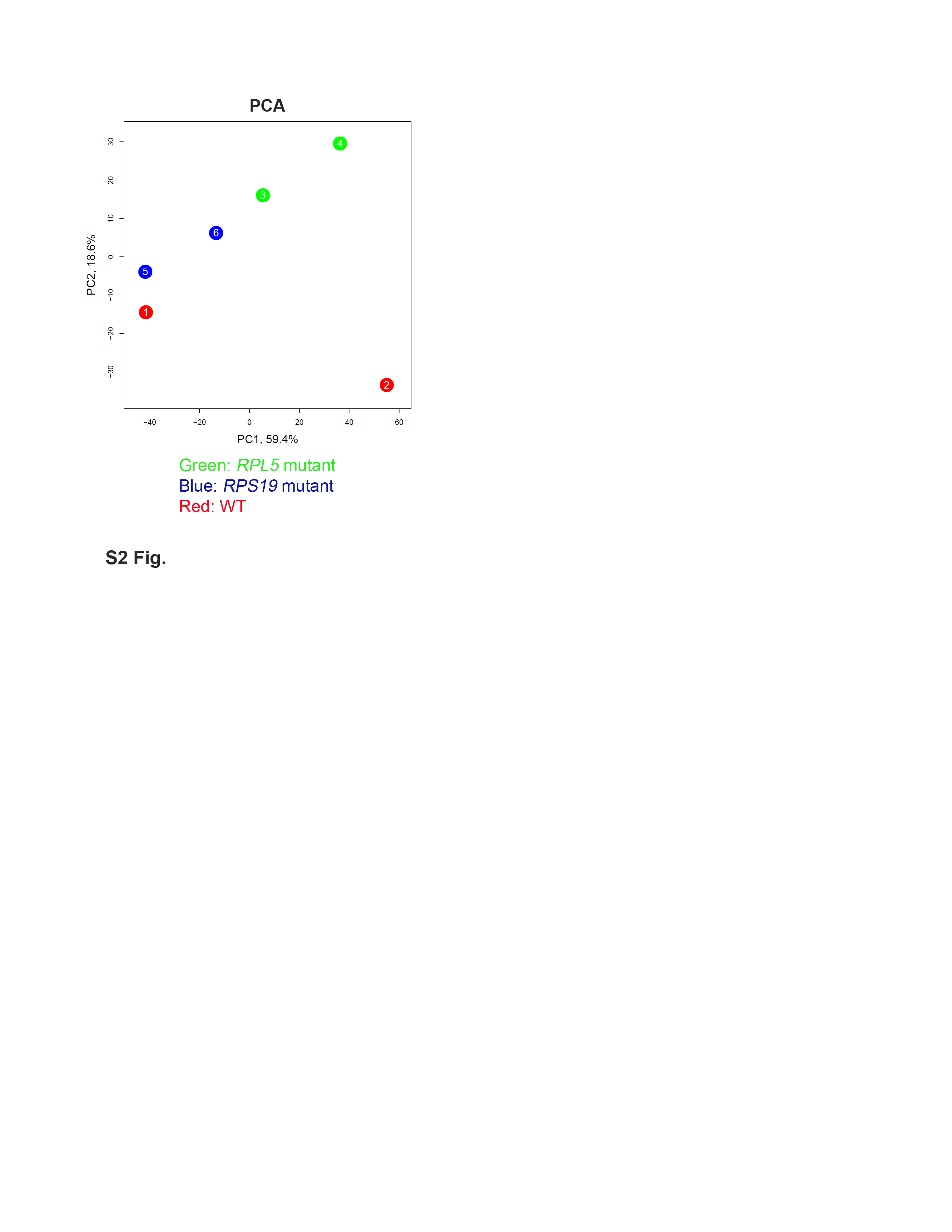

Supplement: S2 Fig — RNA from iPSCs was used for Affymetrix Genechip human exon microarray, and PCA was performed to show overall gene expression difference between DBA mutant iPSCs and wild type iPSCs. (TIF) [file pone.0134878.s002.tif]

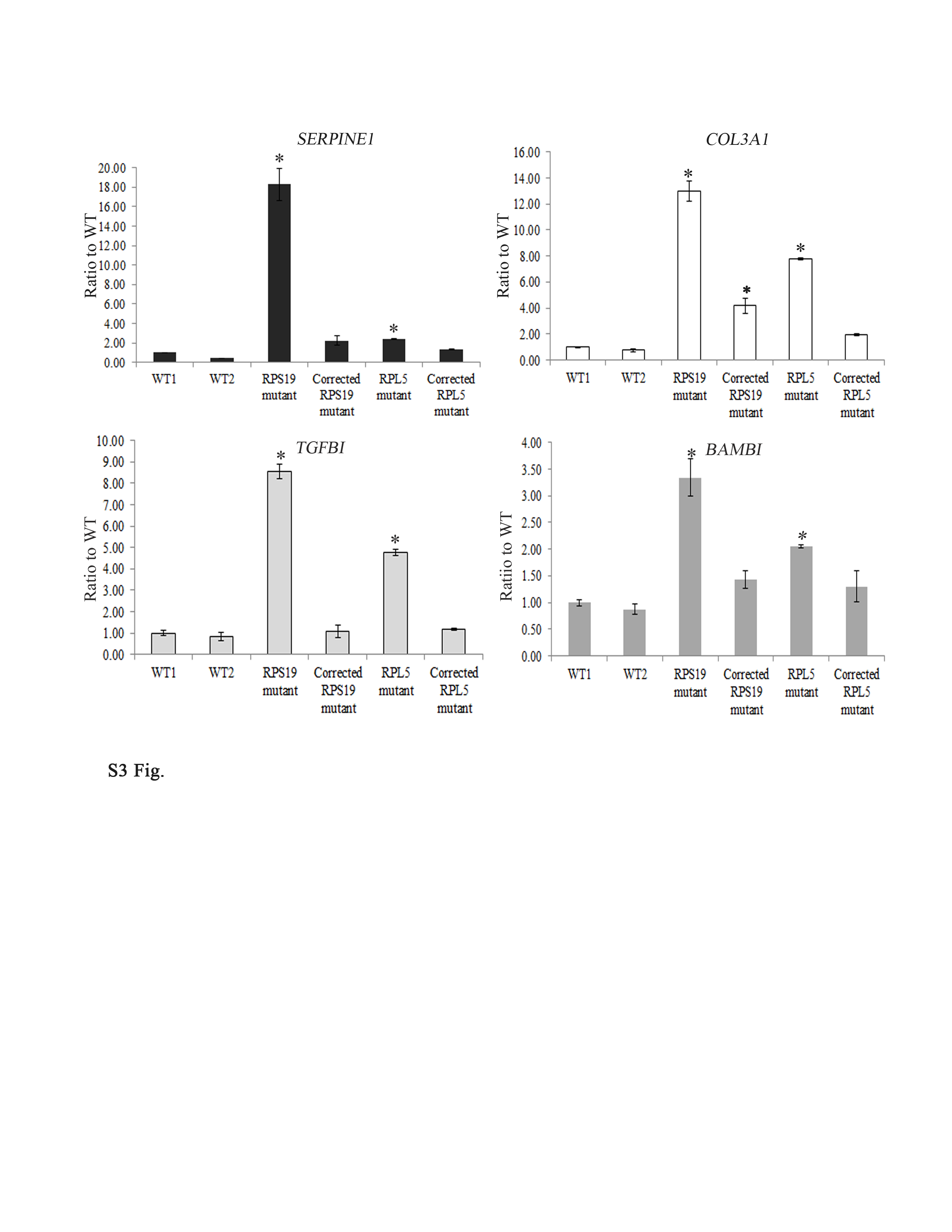

Supplement: S3 Fig — DBA iPSCs were cultured in iPSC medium for 2 days to obtain a homogeneous population of undifferentiated iPSCs, and RNA was extracted for q-PCR. The TGFβ down-stream genes, such as PAI-1, COL3A1, TGFBI, and BAMBI, were measured in DBA iPSCs with RPS19 or RLP5 mutations, and we observed a significant increase in the DBA iPSCs compared to the levels in the wild-type cells. The corrected lines showed a drastic decrease compared to mutant lines. *p<0.05 compared to wildtype. (TIF) [file pone.0134878.s003.tif]

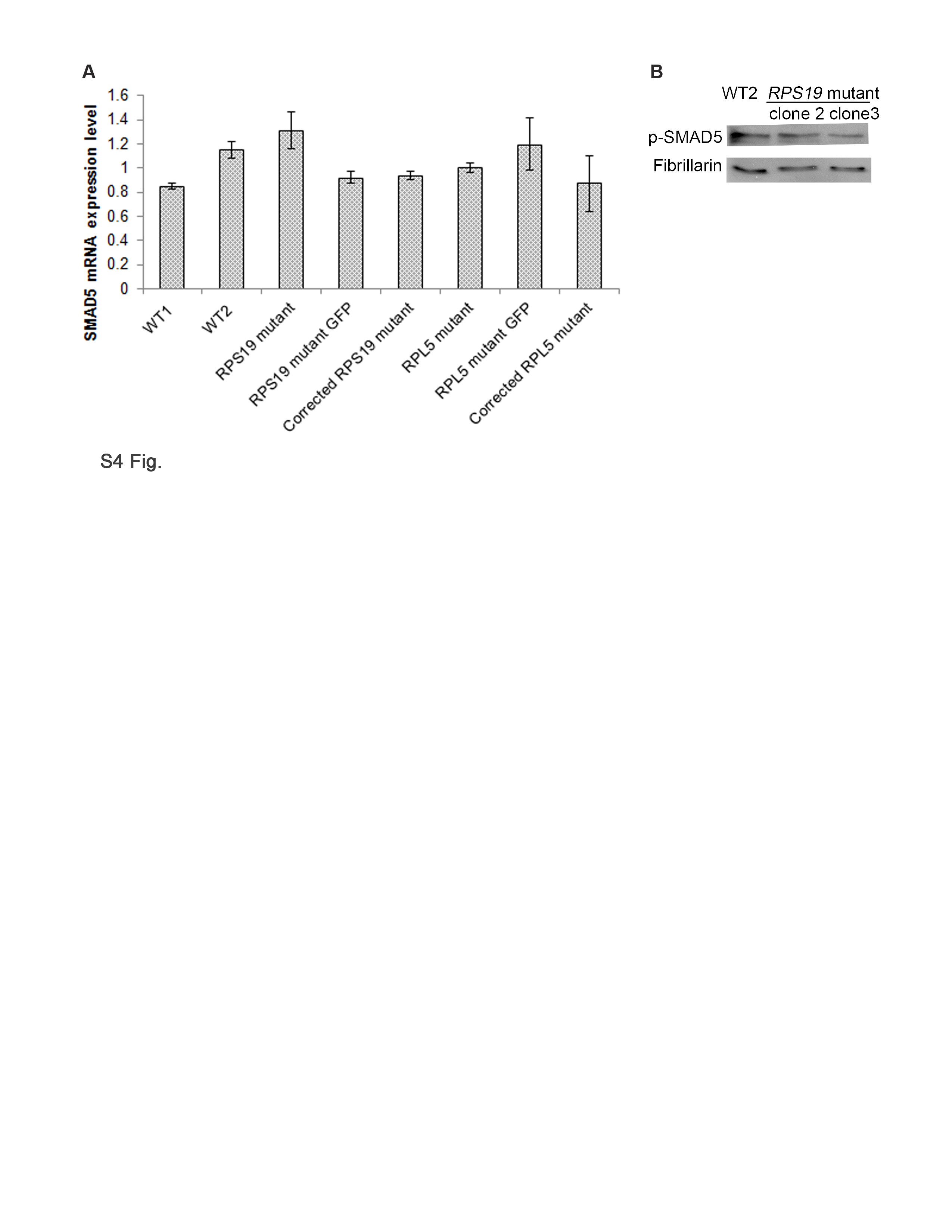

Supplement: S4 Fig — DBA iPSCs were cultured in iPSC medium for 2 days. RNA and protein was extracted for q-PCR and western blot. A) No change of SMAD5 mRNA level in DBA iPSCs with RPS19 or RPL5 mutations. B) Western blot showed no significant change of nuclear p-SMAD5 level in two DBA lines with the same RPS19 mutation from different patients. (TIF) [file pone.0134878.s004.tif]

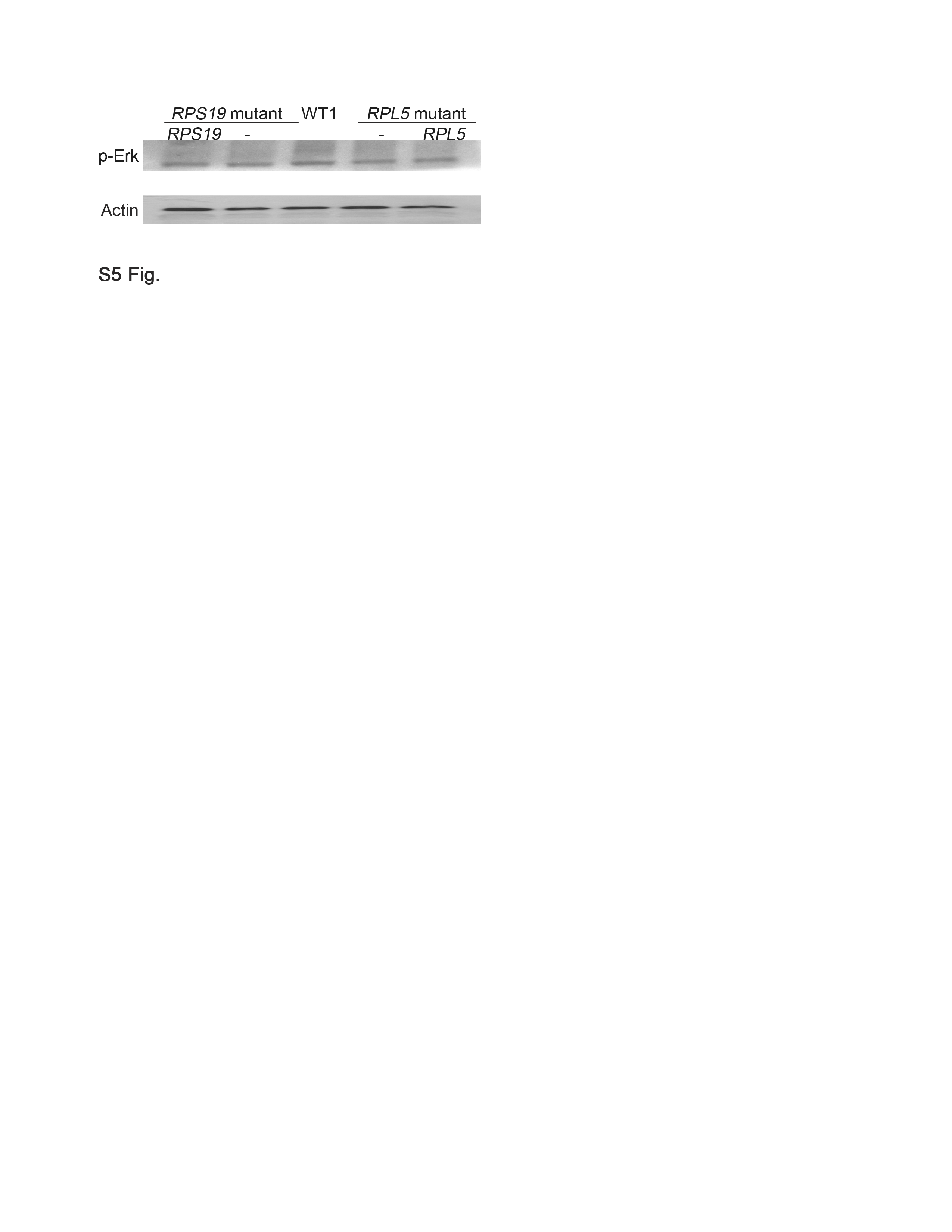

Supplement: S5 Fig — DBA iPSCs were cultured in iPSC medium for 2 days, and protein was extracted for western blot. We observed no change of p-Erk protein in DBA cells compared to wild type cells. (TIF) [file pone.0134878.s005.tif]

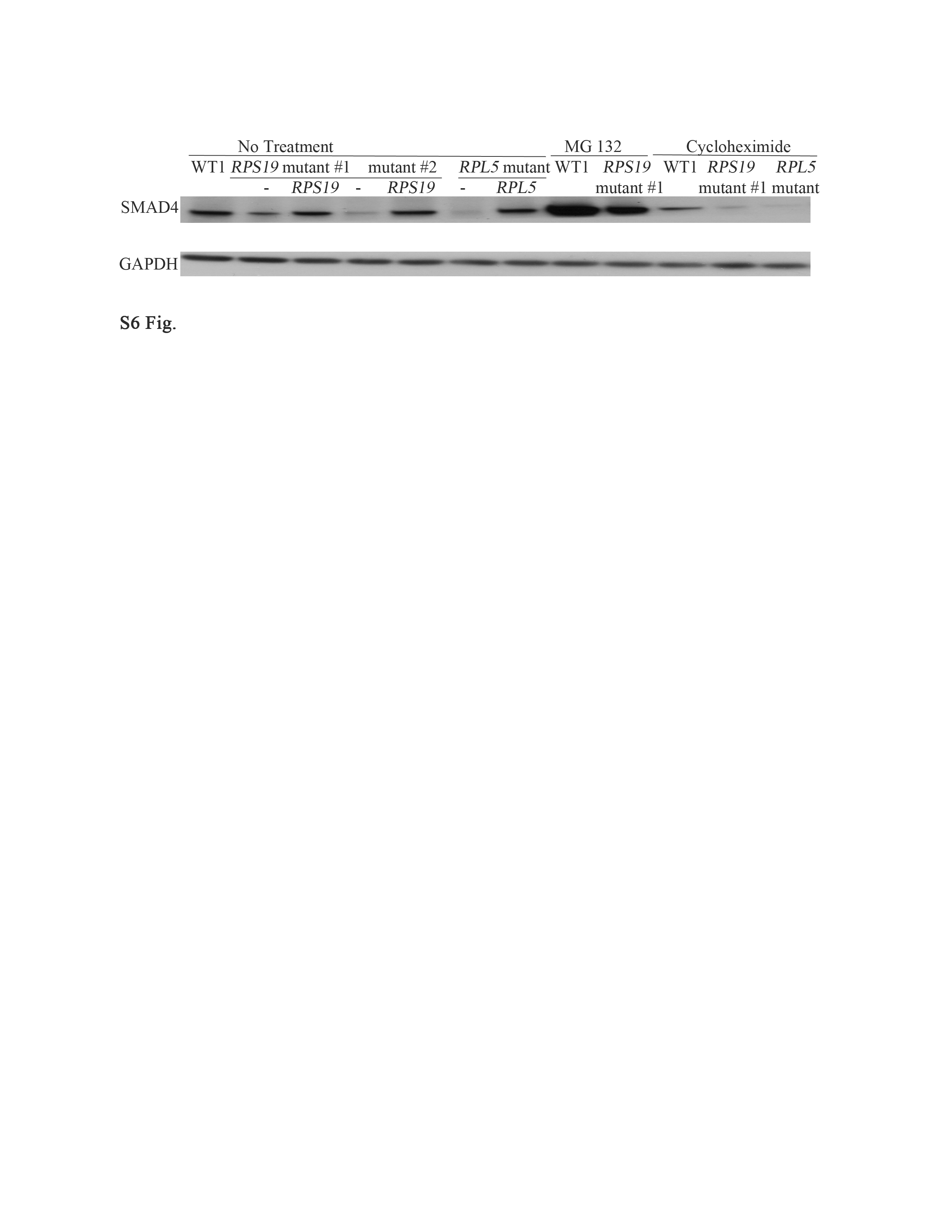

Supplement: S6 Fig — DBA iPSCs were cultured in iPSC medium for 2 days, and protein was extracted for western blot. Treatment with protein synthesis inhibitor cycloheximide decreased the SMAD4 protein level in DBA and WT iPSCs, but did not change the ratio of SMAD4 protein between DBA and wild type cells. The gel also shows the effect of MG132 on SMAD4 levels in the RPS19 mutant cell line which are also the subject of Fig 5B. (TIF) [file pone.0134878.s006.tif]

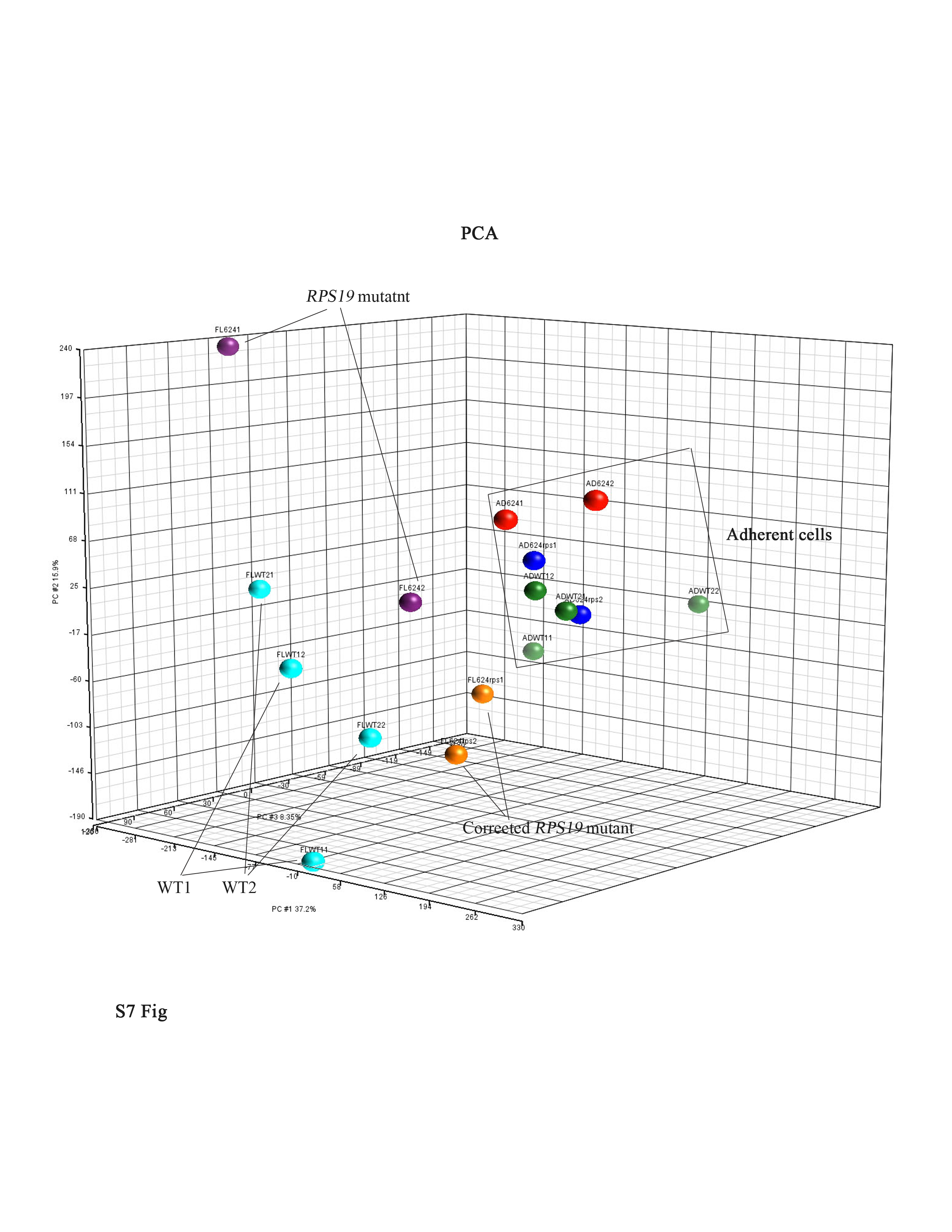

Supplement: S7 Fig — iPSCs were differentiated to EPCs as described by Paluru et al. 25., RNA from derived multilineage progenitors on Day 8 was used for Affymetrix Genechip human transcriptome microarray. PCA was performed to show overall gene expression difference among DBA cells, corrected cells and wild type cells. (TIF) [file pone.0134878.s007.tif]
