# Supplementary material for: Dysregulation of the Transforming Growth Factor β Pathway in Induced Pluripotent Stem Cells Generated from Patients with Diamond Blackfan Anemia
Source: PLoS One. 2015 Aug 10;10(8):e0134878. doi: 10.1371/journal.pone.0134878 (PMC4530889; doi:10.1371/journal.pone.0134878)
Supplement: S1 Table — (DOCX) [file pone.0134878.s008.docx]

**S1Table. Primers used for q-PCR**

| Primer ID | Primer sequence |
| --- | --- |
| CDKN21 F | GAG GCC GGG ATG AGT TGG GAG GAG |
| CDKN21 R | CAG CCG GCG TTT GGA GTG GTA GAA |
| GATA1 F | TGG AGA CTT TGA AGA CAG AGC GGC TGA G |
| GATA1 R | GAA GCT TGG GAG AGG AAT AGG CTG CTG A |
| TGFβ2 F | AAA GCC AGA GTG CCT GAA CA |
| TGFβ2 R | AGC GCT GGG TTG GAG ATG |
| SMAD4 F | TAC CAG CAC TGC CAA CTT TCC |
| SMAD4 R | TGC TAT CTG CAA CAG TCC TTC ACT AT |
| TGFBI F | TTT GTT TAT CGT AAT AGC CTC TGC ATT |
| TGFBI R | CCG TAC CTC CCC CTC TTG TC |
| COL3A1 1F | CCC ACT ATT ATT TTG GCA CAA CAG |
| COL3A1 1R | AAC GGA TCC TGA GTC ACA GAC A |
| BAMBI F | GCT GTC ATG AAG ACA TGT GCA AT |
| BAMBI R | CCA TCA TGC TGA TAC CTG TTT CC |
| SERPINE1 F | CAT TCC ACG TTC TTA ACT GTT CCA |
| SERPINE1 R | GGG CAA AG GAA AAA AAT GAG |
| TGFb1R F | GCA TGG ATC CCT TTT TGA TTA CTT |
| TGFb1R R | CCA TGT GAA GAT GGG CAA GA |
| GAPDH F | AAT CCC ATC ACC ATC TTC CA |
| GAPDH R | TGG ACT CCA CGA CGT ACT CA |
