# Supplementary material for: Dysregulation of the Transforming Growth Factor β Pathway in Induced Pluripotent Stem Cells Generated from Patients with Diamond Blackfan Anemia
Source: PLoS One. 2015 Aug 10;10(8):e0134878. doi: 10.1371/journal.pone.0134878 (PMC4530889; doi:10.1371/journal.pone.0134878)
Supplement: S2 Table — (DOCX) [file pone.0134878.s009.docx]

**S2 Table. Antibodies that were used in the flow cytometry**

| **Antibody ID** | **Category** | **Manufacture** | **Dilution** |
| --- | --- | --- | --- |
| SSEA3 AlexaFluor488 | Pluripotency Markers | Biolegend (San Diego, CA) | 1:100 |
| SSEA 4 AlexaFluor 647 | Pluripotency Markers | Biolegend (San Diego, CA) | 1:100 |
| TRA 1-61 AlexaFluor 488 | Pluripotency Markers | Biolegend (San Diego, CA) | 1:20 |
| TRA 1-81 AlexaFluor 647 | Pluripotency Markers | Biolegend (San Diego, CA) | 1:50 |
| KDR/VEGF R2 PE | Differentiation markers for adherent cells | R&D Systems (Minneapolis, MN) | 1:20 |
| CD31 AlexaFluor 488 | Differentiation markers for adherent cells | BD Bioscience (San Jose, CA) | 1:20 |
| CD117 APC | Differentiation markers for adherent cells | Biolegend (San Diego, CA) | 1:20 |
| CD41a APC | Differentiation markers for adherent cells | Biolegend (San Diego, CA) | 1:100 |
| CD43 FITC | Differentiation markers for floating cells | Biolegend (San Diego, CA) | 1:50 |
| CD41a PE | Differentiation markers for floating cells | BD Bioscience (San Jose, CA) | 1:50 |
| CD34 APC | Differentiation markers for floating cells | CALTAG Lab (Buckingham, UK) | 1:100 |
| CD235a APC | Differentiation markers for floating cells | BD Bioscience (San Jose, CA) | 1:10000 |
