# Supplementary material for: Dysregulation of the Transforming Growth Factor β Pathway in Induced Pluripotent Stem Cells Generated from Patients with Diamond Blackfan Anemia
Source: PLoS One. 2015 Aug 10;10(8):e0134878. doi: 10.1371/journal.pone.0134878 (PMC4530889; doi:10.1371/journal.pone.0134878)
Supplement: S3 Table — (DOCX) [file pone.0134878.s010.docx]

**S3 Table. Common genes whose expression differed by 2-fold or more between DBA iPSCs with *RPS19* or *RPL5* mutations and wild type iPSCs**

|  |  | **Fold change** | |  |
| --- | --- | --- | --- | --- |
| **Symbol** | **Entrez Gene Name** | ***RPS19*** | ***RPL5*** | **p** |
| CER1 | cerberus 1, cysteine knot superfamily, homolog (Xenopus laevis) | 18.3595 | 2.1427 | 0.001 |
| CST1 | cystatin SN | 14.5832 | 32.4722 | 0.000 |
| SNORD41 | small nucleolar RNA, C/D box 41 | 10.4311 | 9.3632 | 0.048 |
| PRTG | protogenin | 9.0413 | 13.9486 | 0.000 |
| LOC441666 | zinc finger protein 91 pseudogene | 7.6265 | 0.2977 | 0.048 |
| CRYZ | crystallin, zeta (quinone reductase) | 7.5742 | 8.5188 | 0.000 |
| DKK1 | dickkopf homolog 1 (Xenopus laevis) | 7.1095 | 5.3811 | 0.000 |
| TYW3 | tRNA-yW synthesizing protein 3 homolog (S. cerevisiae) | 6.7472 | 7.1207 | 0.000 |
| NTS | neurotensin | 6.3254 | 3.4499 | 0.018 |
| DUSP6 | dual specificity phosphatase 6 | 6.1710 | 5.1470 | 0.000 |
| RPS4Y1 | ribosomal protein S4, Y-linked 1 | 5.9634 | 5.4883 | 0.005 |
| IGFBP5 | insulin-like growth factor binding protein 5 | 5.1716 | 3.1784 | 0.001 |
| HAS2 | hyaluronan synthase 2 | 5.1173 | 4.3948 | 0.000 |
| TNC | tenascin C | 4.7120 | 3.3950 | 0.000 |
| NLGN4Y | neuroligin 4, Y-linked | 4.6726 | 3.2457 | 0.022 |
| CDH6 | cadherin 6, type 2, K-cadherin (fetal kidney) | 4.4912 | 8.8203 | 0.000 |
| SNAI2 | snail homolog 2 (Drosophila) | 4.4689 | 7.2485 | 0.000 |
| TGFBI | transforming growth factor, beta-induced, 68kDa | 4.4409 | 3.7050 | 0.000 |
| GRPR | gastrin-releasing peptide receptor | 4.4122 | 3.3969 | 0.001 |
| RHOBTB3 | Rho-related BTB domain containing 3 | 3.8950 | 3.2209 | 0.000 |
| LGI1 | leucine-rich, glioma inactivated 1 | 3.7132 | 3.2825 | 0.002 |
| FN1 | fibronectin 1 | 3.6765 | 3.1632 | 0.000 |
| GLIPR1 | GLI pathogenesis-related 1 | 3.6614 | 0.4924 | 0.019 |
| GPC6 | glypican 6 | 3.5311 | 2.7733 | 0.000 |
| KDM5D | lysine (K)-specific demethylase 5D | 3.5310 | 2.7929 | 0.033 |
| MIR125A | microRNA 125a | 3.5089 | 2.2226 | 0.000 |
| FBN2 | fibrillin 2 | 3.4882 | 2.3264 | 0.000 |
| LGR5 | leucine-rich repeat containing G protein-coupled receptor 5 | 3.4633 | 5.2435 | 0.000 |
| TAGLN | transgelin | 3.4474 | 3.4629 | 0.000 |
| WLS | wntless homolog (Drosophila) | 3.2848 | 3.3917 | 0.000 |
| ZEB1 | zinc finger E-box binding homeobox 1 | 3.0577 | 2.1224 | 0.002 |
| MMP2 | matrix metallopeptidase 2 (gelatinase A, 72kDa gelatinase, 72kDa type IV collagenase) | 3.0193 | 2.5603 | 0.002 |
| GPR50 | G protein-coupled receptor 50 | 2.9699 | 2.8841 | 0.000 |
| FBN1 | fibrillin 1 | 2.9540 | 2.7590 | 0.001 |
| CDH2 | cadherin 2, type 1, N-cadherin (neuronal) | 2.9525 | 3.9191 | 0.000 |
| USP3 | ubiquitin specific peptidase 3 | 2.9336 | 2.2477 | 0.001 |
| PRRX1 | paired related homeobox 1 | 2.8433 | 2.1810 | 0.003 |
| PCDH10 | protocadherin 10 | 2.7716 | 2.1355 | 0.001 |
| FLRT3 | fibronectin leucine rich transmembrane protein 3 | 2.7283 | 2.0952 | 0.004 |
| LUM | lumican | 2.7234 | 6.3409 | 0.000 |
| FLRT2 | fibronectin leucine rich transmembrane protein 2 | 2.7102 | 4.4462 | 0.000 |
| COLEC12 | collectin sub-family member 12 | 2.7049 | 2.1377 | 0.001 |
| LOC100132832 | postmeiotic segregation increased 2-like 5-like | 2.6616 | 0.4811 | 0.023 |
| SERPINE1 | serpin peptidase inhibitor, clade E (nexin, plasminogen activator inhibitor type 1), member 1 | 2.6197 | 2.1121 | 0.001 |
| PLAT | plasminogen activator, tissue | 2.6094 | 3.0426 | 0.000 |
| TMEM88 | transmembrane protein 88 | 2.5990 | 2.3539 | 0.000 |
| NCRNA00261 | non-protein coding RNA 261 | 2.5780 | 2.6843 | 0.000 |
| EOMES | eomesodermin | 2.5740 | 2.3420 | 0.001 |
| CDH11 | cadherin 11, type 2, OB-cadherin (osteoblast) | 2.5611 | 2.0739 | 0.016 |
| F2RL2 | coagulation factor II (thrombin) receptor-like 2 | 2.5066 | 3.1701 | 0.002 |
| GRID2 | glutamate receptor, ionotropic, delta 2 | 2.4031 | 2.2382 | 0.001 |
| ELMO1 | engulfment and cell motility 1 | 2.3886 | 2.1064 | 0.002 |
| GREM1 | gremlin 1 | 2.3618 | 2.1960 | 0.001 |
| SNORD35A | small nucleolar RNA, C/D box 35A | 2.3414 | 2.0706 | 0.031 |
| CSRP1 | cysteine and glycine-rich protein 1 | 2.3189 | 2.3042 | 0.001 |
| IFI16 | interferon, gamma-inducible protein 16 | 2.3103 | 2.0363 | 0.011 |
| LIX1 | Lix1 homolog (chicken) | 2.2939 | 2.7245 | 0.008 |
| MIXL1 | Mix1 homeobox-like 1 (Xenopus laevis) | 2.2894 | 2.1798 | 0.001 |
| ADAMTS9 | ADAM metallopeptidase with thrombospondin type 1 motif, 9 | 2.2557 | 4.7502 | 0.000 |
| GREB1L | growth regulation by estrogen in breast cancer-like | 2.2400 | 2.7265 | 0.002 |
| FRZB | frizzled-related protein | 2.1730 | 3.7284 | 0.000 |
| LLPH | LLP homolog, long-term synaptic facilitation (Aplysia) | 2.1390 | 2.4910 | 0.009 |
| THY1 | Thy-1 cell surface antigen | 2.1177 | 2.0941 | 0.008 |
| NODAL | nodal homolog (mouse) | 2.1099 | 2.4199 | 0.001 |
| ITGA5 | integrin, alpha 5 (fibronectin receptor, alpha polypeptide) | 2.1066 | 2.3341 | 0.000 |
| ALOX12P2 | arachidonate 12-lipoxygenase pseudogene 2 | 2.0987 | 3.6421 | 0.000 |
| MIR1251 | microRNA 1251 | 2.0881 | 2.0801 | 0.000 |
| MAGED2 | melanoma antigen family D, 2 | 2.0799 | 2.1878 | 0.045 |
| AHNAK | AHNAK nucleoprotein | 2.0663 | 2.0565 | 0.001 |
| RGS5 | regulator of G-protein signaling 5 | 2.0309 | 3.8858 | 0.000 |
| SLC2A3 | solute carrier family 2 (facilitated glucose transporter), member 3 | 2.0127 | 2.0015 | 0.016 |
| S1PR3 | sphingosine-1-phosphate receptor 3 | 2.0049 | 2.4365 | 0.000 |
| GK3P | glycerol kinase 3 pseudogene | 0.4839 | 2.1361 | 0.008 |
| PLP1 | proteolipid protein 1 | 0.4413 | 0.4741 | 0.001 |
| ZNF506 | zinc finger protein 506 | 0.3751 | 0.4857 | 0.012 |
| HHLA1 | HERV-H LTR-associating 1 | 0.3599 | 0.4473 | 0.001 |
| GLT1D1 | glycosyltransferase 1 domain containing 1 | 0.3489 | 2.4379 | 0.000 |
| HLA-DPA1 | major histocompatibility complex, class II, DP alpha 1 | 0.3394 | 0.4595 | 0.001 |
| ZNF229 | zinc finger protein 229 | 0.3344 | 0.3774 | 0.006 |
| PKIB | protein kinase (cAMP-dependent, catalytic) inhibitor beta | 0.3283 | 0.4583 | 0.013 |
| MX2 | myxovirus (influenza virus) resistance 2 (mouse) | 0.3113 | 0.3323 | 0.029 |
| NNAT | neuronatin | 0.0579 | 2.7979 | 0.003 |
| ZFP42 | zinc finger protein 42 homolog (mouse) | 0.0290 | 0.4546 | 0.011 |
