# Supplementary material for: Dysregulation of the Transforming Growth Factor β Pathway in Induced Pluripotent Stem Cells Generated from Patients with Diamond Blackfan Anemia
Source: PLoS One. 2015 Aug 10;10(8):e0134878. doi: 10.1371/journal.pone.0134878 (PMC4530889; doi:10.1371/journal.pone.0134878)
Supplement: S4 Table — (DOCX) [file pone.0134878.s011.docx]

| **Category** | **Count** | **%** | **p value** | **Fold** | **Genes** |
| --- | --- | --- | --- | --- | --- |
| Focal adhesion | 21 | 5.290 | 0.000 | 3.689 | TNC, ITGA1, ITGA3, HGF, MAPK10, VAV1, COL5A2, MYL9, VCL, LAMA4, DOCK1, ITGA5, ITGA8, COL6A3, COL1A2, COL1A1, SHC3, THBS1, EGF, PIK3R1, FN1 |
| ECM-receptor interaction | 12 | 3.023 | 0.000 | 5.045 | LAMA4, ITGA5, TNC, ITGA8, COL6A3, COL1A2, ITGA1, ITGA3, COL1A1, THBS1, COL5A2, FN1 |
| Wnt signaling pathway | 10 | 2.519 | 0.026 | 2.339 | CER1, DKK1, EP300, LEF1, WIF1, MAPK10, PPP3CA, FZD2, PLCB2, DKK4 |
| Calcium signaling pathway | 11 | 2.771 | 0.026 | 2.207 | EDNRA, EDNRB, TACR3, CCKBR, ADORA2A, HTR7, GRPR, RYR2, PPP3CA, PLCB2, ITPR2 |
| Type I diabetes mellitus | 5 | 1.259 | 0.029 | 4.204 | CPE, HLA-DRB5, HLA-DPA1, HLA-DPB1, GAD1 |
| Pathways in cancer | 16 | 4.030 | 0.041 | 1.723 | MMP9, LEF1, ITGA3, KIT, FZD2, HGF, MAPK10, MMP2, CTNNA3, MMP1, LAMA4, EP300, EGF, CCNA1, PIK3R1, FN1 |
| Hematopoietic cell lineage | 6 | 1.511 | 0.019 | 2.464 | CR2, ITGA5, ITGA1, HLA-DRB5, ITGA3, KIT |
| TGF beta signaling pathway | 6 | 1.511 | 0.030 | 2.435 | NOG, EP300, ID2, NODAL, FST, THBS1 |
| Leukocyte transendothelial migration | 9 | 2.267 | 0.017 | 2.693 | MMP9, CLDN11, VAV1, MMP2, CTNNA3, PIK3R1, VCL, THY1, MYL9 |
| Apoptosis | 6 | 1.511 | 0.047 | 2.435 | PRKAR2B, TNFRSF10C, TNFRSF10B, TNFRSF10D, PPP3CA, PIK3R1 |

**S4 Table. David pathway analysis of iPSCs with *RPS19* mutation.**
