# Supplementary material for: Dysregulation of the Transforming Growth Factor β Pathway in Induced Pluripotent Stem Cells Generated from Patients with Diamond Blackfan Anemia
Source: PLoS One. 2015 Aug 10;10(8):e0134878. doi: 10.1371/journal.pone.0134878 (PMC4530889; doi:10.1371/journal.pone.0134878)
Supplement: S5 Table — (DOCX) [file pone.0134878.s012.docx]

**S5 Table. David pathway analysis summary of iPSCs with *RPL5* mutation.**

| **Category** | **Count** | **%** | **p value** | **Fold** | **Genes in net work** |
| --- | --- | --- | --- | --- | --- |
| Focal adhesion | 25 | 2.867 | 0.000 | 2.750 | TNC, COL3A1, PAK2, SOS1, COL6A3, THBS1, EGF, SHC3, FIGF, AKT3, PIK3R1, FN1, ACTB, COL4A2, COL4A1, ROCK1, ITGA3, HGF, COL5A2, ITGA6, ITGA5, COL1A2, RAP1B, COL1A1, PTENP1 |
| ECM-receptor interaction | 14 | 1.606 | 0.000 | 3.685 | COL4A2, COL4A1, TNC, COL3A1, ITGA3, COL5A2, HMMR, ITGA6, ITGA5, COL6A3, COL1A2, COL1A1, THBS1, FN1 |
| Renal cell carcinoma | 10 | 1.147 | 0.004 | 3.158 | CUL2, PAK2, EPAS1, SOS1, RAP1B, HGF, FIGF, AKT3, TGFB1, PIK3R1 |
| Systemic lupus erythematosus | 12 | 1.376 | 0.005 | 2.680 | HIST1H2BC, HIST1H2BK, HLA-DRB1, HIST2H2BF, HIST1H2BJ, HLA-DRB5, HIST1H4F, HLA-DPA1, HIST1H2AJ, HLA-DPB1, HLA-DOA, HIST3H2BB |
| Allograft rejection | 5 | 0.573 | 0.008 | 3.071 | HLA-DRB1, HLA-DRB5, HLA-DPA1, HLA-DPB1, HLA-DOA |
| mTOR signaling pathway | 6 | 0.688 | 0.008 | 2.551 | EIF4B, RPS6KA6, IGF2, FIGF, AKT3, PIK3R1 |
| TGF beta signaling pathway | 8 | 0.917 | 0.010 | 2.033 | NOG, ID2, ROCK1, E2F5, NODAL, FST, THBS1, TGFB1 |
| Graft-versus-host disease | 5 | 0.573 | 0.020 | 2.834 | HLA-DRB1, HLA-DRB5, HLA-DPA1, HLA-DPB1, HLA-DOA |
| Viral myocarditis | 7 | 0.803 | 0.020 | 2.180 | ACTB, HLA-DRB1, HLA-DRB5, HLA-DPA1, HLA-DPB1, HLA-DOA, CXADR |
| Pathways in cancer | 21 | 2.408 | 0.100 | 1.415 | WNT5A, COL4A2, COL4A1, EPAS1, MMP9, LEF1, ITGA3, RB1, FZD2, HGF, MMP2, TGFB1, CUL2, ITGA6, SOS1, EGF, FIGF, PTENP1, PIK3R1, AKT3, FN1 |
