# Supplementary material for: Dysregulation of the Transforming Growth Factor β Pathway in Induced Pluripotent Stem Cells Generated from Patients with Diamond Blackfan Anemia
Source: PLoS One. 2015 Aug 10;10(8):e0134878. doi: 10.1371/journal.pone.0134878 (PMC4530889; doi:10.1371/journal.pone.0134878)
Supplement: S6 Table — (DOCX) [file pone.0134878.s013.docx]

**S6 Table. Genes were significantly changed in DBA cells with *RPS19* mutation compared with the wild-type and restored in the correct lines at day 8 differentiation.**

| **Symbol** | **Entrez Gene Name** | **DBA vs WT** | | **Corrected vs DBA** | |
| --- | --- | --- | --- | --- | --- |
|  |  | Fold | p | Fold | p |
| SNORD93 | small nucleolar RNA, C/D box 93 | -9.244 | 0.000 | -2.561 | 0.047 |
| ITGA4 | integrin, alpha 4 (antigen CD49D, alpha 4 subunit of VLA-4 receptor) | -8.986 | 0.000 | -2.436 | 0.024 |
| PIK3CG | phosphatidylinositol-4,5-bisphosphate 3-kinase, catalytic subunit gamma | -6.379 | 0.001 | -3.083 | 0.024 |
| MNS1 | meiosis-specific nuclear structural 1 | -5.221 | 0.000 | -3.241 | 0.002 |
| ZNF562 | zinc finger protein 562 | -5.166 | 0.000 | -2.848 | 0.000 |
| KIT | v-kit Hardy-Zuckerman 4 feline sarcoma viral oncogene homolog | -4.751 | 0.004 | -3.403 | 0.027 |
| FAR2 | fatty acyl CoA reductase 2 | -4.486 | 0.003 | -4.728 | 0.007 |
| SLCO4C1 | solute carrier organic anion transporter family, member 4C1 | -4.365 | 0.005 | -3.331 | 0.030 |
| HIST1H2AB | histone cluster 1, H2ab | -4.213 | 0.000 | -2.602 | 0.005 |
| CTSC | cathepsin C | -4.004 | 0.007 | -3.307 | 0.029 |
| ARHGDIB | Rho GDP dissociation inhibitor (GDI) beta | -3.72 | 0.001 | -2.248 | 0.026 |
| FKBP5 | FK506 binding protein 5 | -3.352 | 0.001 | -2.471 | 0.009 |
| CDCA7L | cell division cycle associated 7-like | -3.265 | 0.009 | -3.656 | 0.012 |
| CYYR1 | cysteine/tyrosine-rich 1 | -2.859 | 0.009 | -3.321 | 0.010 |
| SLFN13 | schlafen family member 13 | -2.683 | 0.000 | -2.018 | 0.000 |
| VAV3 | vav 3 guanine nucleotide exchange factor | -2.524 | 0.015 | -2.809 | 0.018 |
| SYT11 | synaptotagmin XI | -2.523 | 0.006 | -2.032 | 0.044 |
| SPAG7 | sperm associated antigen 7 | -2.368 | 0.000 | -2.415 | 0.000 |
| CENPV | centromere protein V | -2.361 | 0.001 | -2.144 | 0.004 |
| EYA2 | eyes absent homolog 2 (Drosophila) | 2.08 | 0.007 | 2.109 | 0.015 |
| AHSG | alpha-2-HS-glycoprotein | 2.097 | 0.011 | 2.079 | 0.024 |
| NEXN | nexilin (F actin binding protein) | 2.1 | 0.002 | 2.001 | 0.008 |
| SPNS2 | spinster homolog 2 (Drosophila) | 2.14 | 0.008 | 2.542 | 0.006 |
| LCN9 | lipocalin 9 | 2.384 | 0.001 | 2.484 | 0.003 |
| MACC1 | metastasis associated in colon cancer 1 | 2.404 | 0.022 | 2.643 | 0.026 |
| SLC1A3 | solute carrier family 1 (glial high affinity glutamate transporter), member 3 | 2.428 | 0.006 | 2.359 | 0.016 |
| TMEM178B | transmembrane protein 178B | 2.525 | 0.002 | 2.839 | 0.002 |
| ANXA3 | annexin A3 | 2.607 | 0.043 | 4.328 | 0.012 |
| PCSK5 | proprotein convertase subtilisin/kexin type 5 | 2.717 | 0.015 | 2.592 | 0.036 |
| OR2A4/OR2A7 | olfactory receptor, family 2, subfamily A, member 4 | 2.753 | 0.003 | 2.136 | 0.030 |
| GPR87 | G protein-coupled receptor 87 | 2.791 | 0.008 | 3.178 | 0.009 |
| HOOK1 | hook microtubule-tethering protein 1 | 2.793 | 0.008 | 2.446 | 0.033 |
| INADL | InaD-like (Drosophila) | 2.804 | 0.018 | 2.61 | 0.046 |
| FAM84B | family with sequence similarity 84, member B | 2.843 | 0.013 | 3.635 | 0.009 |
| TFAP2A | transcription factor AP-2 alpha (activating enhancer binding protein 2 alpha) | 2.894 | 0.015 | 3.01 | 0.025 |
| BNC1 | basonuclin 1 | 2.9 | 0.023 | 3.068 | 0.035 |
| RASGRF2 | Ras protein-specific guanine nucleotide-releasing factor 2 | 2.91 | 0.017 | 3.172 | 0.023 |
| CCBE1 | collagen and calcium binding EGF domains 1 | 3.078 | 0.035 | 3.448 | 0.042 |
| ADAMTS6 | ADAM metallopeptidase with thrombospondin type 1 motif, 6 | 3.223 | 0.016 | 3.395 | 0.026 |
| TNFRSF19 | tumor necrosis factor receptor superfamily, member 19 | 3.232 | 0.002 | 2.98 | 0.007 |
| TNFSF15 | tumor necrosis factor (ligand) superfamily, member 15 | 3.4 | 0.008 | 4.299 | 0.007 |
| INHBA | inhibin, beta A | 3.435 | 0.000 | 3.941 | 0.000 |
| GRHL2 | grainyhead-like 2 (Drosophila) | 4.156 | 0.023 | 4.381 | 0.037 |
| CDON | cell adhesion associated, oncogene regulated | 4.316 | 0.015 | 4.152 | 0.033 |
| PRRG4 | proline rich Gla (G-carboxyglutamic acid) 4 (transmembrane) | 4.9 | 0.008 | 5.48 | 0.012 |
| FBXL21 | F-box and leucine-rich repeat protein 21 (gene/pseudogene) | 6.302 | 0.037 | 8.766 | 0.034 |
| CALB2 | calbindin 2 | 7.684 | 0.000 | 8.635 | 0.000 |
| SPTLC3 | serine palmitoyltransferase, long chain base subunit 3 | 8.815 | 0.002 | 8.619 | 0.005 |
