# Supplementary material for: Dysregulation of the Transforming Growth Factor β Pathway in Induced Pluripotent Stem Cells Generated from Patients with Diamond Blackfan Anemia
Source: PLoS One. 2015 Aug 10;10(8):e0134878. doi: 10.1371/journal.pone.0134878 (PMC4530889; doi:10.1371/journal.pone.0134878)
Supplement: S7 Table — (DOCX) [file pone.0134878.s014.docx]

**S7 Table. Ingenuity analysis of pathways and molecular and cellular functions in hematopoietic progenitors with *RPS19* mutation on day 8.**

| **Pathways** | **p-value** | **Ratio** |
| --- | --- | --- |
| Tec Kinase Signaling | 4.12E-09 | 27/148 |
| Virus Entry via Endocytic Pathways | 1.08E-08 | 20/89 |
| Leukocyte Extravasation Signaling | 8.94E-08 | 29/192 |
| Thrombopoietin Signaling | 3.37E-07 | 14/55 |
| Cell Cycle Control of Chromosomal Replication | 3.48E-07 | 10/27 |
|  |  |  |
| **Molecular and Cellular Functions** | **p-value** | **Molecules** |
| Cellular Growth and Proliferation | 9.39E-04 | 269 |
| Cell Death and Survival | 9.25E-04 | 260 |
| Cell Cycle | 9.34E-04 | 154 |
| Cellular Movement | 9.99E-04 | 182 |
| Cellular Assembly and Organization | 6.31E-04 | 116 |
