# Supplementary material for: Dysregulation of the Transforming Growth Factor β Pathway in Induced Pluripotent Stem Cells Generated from Patients with Diamond Blackfan Anemia
Source: PLoS One. 2015 Aug 10;10(8):e0134878. doi: 10.1371/journal.pone.0134878 (PMC4530889; doi:10.1371/journal.pone.0134878)
Supplement: S8 Table — (DOCX) [file pone.0134878.s015.docx]

**S8 Table. Ingenuity analysis of upstream regulators in hematopoietic progenitors with *RPS19* mutation on day 8.**

| **Upstream Regulator** | **p-value** | **Predicted State** | **Activation z-score** | | **Target molecules in dataset** |
| --- | --- | --- | --- | --- | --- |
| TGFβ1 | 1.14E-20 | Activated | 2.79 | ADAM19,AFP,AKR1C1/AKR1C2,ANKRD1,ASPM,BRIP1,BUB1,CADM1,CCNA2,CCNB1,CCNB2,CDC25C,CDH1,CDK1,CDKN3,CENPE,CENPF,CKS2,COL1A1,COL6A3,CTGF,EDN1,FBLN1,ITGA2,ITGA3,ITGA4,ITGAV,ITGB3,JUN,LAMC2,MKI67,MMP2,MMP9,MPHOSPH9,MYC,NCAPG,NDC80,NEK2,ORC1,POLE2,PRC1,PRIM1,PRKCA,RAD51AP1,RFC4,SFRP1,SLC39A8,SPARC,TBX3,TGFB1,TGFBR1,TNC,TOP2A,TPM1,WNT5A,ZWINT | |
| E2F4 | 9.24E-20 |  |  | AURKB,BRCA1,CCNA2,CDC25A,CDC6,CDK1,CENPE,CHEK1,CKS2,DBF4,DHFR,HIST2H2AA3/HIST2H2AA4,HMMR,KIAA0101,MAD2L1,MCM10,MCM3,MCM5,MCM6,MKI67,MTHFD1,MYC,NDC80,NEK2,ORC1,PLK1,POLA1,PRC1,PRKDC,RAD51,RAD51AP1,RBL1,RFC3,RFC4,RRM1,RRM2,SPAG7,TOP2A,TTK,UBE2T | |
| NUPR1 | 1.42E-19 | Activated | 5.969 | APOBEC3B,ARHGAP11A,ASPM,ATAD5,ATF3,ATP8B1,AURKA,BLM,BRCA1,BRI3BP,BUB1,CASC5,CCNA2,CCNB2,CCNF,CDC25C,CDCA2,CDCA8,CENPI,CKAP2L,CXADR,CYR61,DNMT3B,E2F8,EXO1,FAM114A1,FAM72C/FAM72D,FANCD2,GK,HIST1H1B,HIST1H2AB,HIST1H2AH,HIST1H2BM,HIST1H3B,HIST1H3F,HJURP,IRS2,ITPR3,KIF11,KIF18A,KIF20A,KIF23,KIF2C,KLF6,LMNB1,MCM10,MKI67,MTFR2,MYC,NDRG1,NEIL3,NSF,OSBPL6,PIM1,PLK1,POLE2,POLQ,RAB38,RAD51,RCL1,RILPL2,SHCBP1,SIPA1L2,SLC16A6,SLC2A1,SLC2A12,SLC39A8,SPATS2L,SPC25,STIL,TFAP2A,TICRR,WDR76,XRCC2 | |
| TP53 | 7.98E-17 | Activated | 4.316 | ATF3,ATG4C,AURKA,AURKB,BRCA1,BUB1,C12orf5,CCNA2,CCNB1,CD47,CDC25A,CDC25C,CDC6,CDC7,CDH1,CDK1,CDKN3,CENPF,CEP55,CHEK1,CHMP4C,CKAP2,CPOX,CTGF,DBF4,DHFR,GSR,HMMR,ITGA2,KIAA0101,KIF23,KRT8,MAD2L1,MCM2,MCM3,MCM6,MCM7,ME2,MMP2,MMP9,MYC,NCAPG,NDC80,NDRG1,NEK2,PARD6B,PBK,PEG10,PERP,PIM1,PLK1,PLK2,POLE2,PRC1,PRIM1,PRKCB,PTPN6,RFC3,RFC4,RRM2,SELP,SEMA3C,SLC2A1,SLC2A12,TGFB1,TJP1,TOP2A,VRK1,WDHD1 | |
| ERBB2 | 6.25E-16 |  | 1.588 | ADAM19,ASPM,BRIP1,BUB1,CADM1,CCNA2,CCNB1,CCNB2,CDC25C,CDK1,CDKN3,CENPE,CENPF,CKS2,COL1A1,COL6A3,CTGF,EDN1,ERBB3,ITGA2,JUN,LAMC2,MKI67,MPHOSPH9,MYC,NCAPG,NDC80,NEK2,POLE2,PRC1,PRIM1,RAD51AP1,RFC4,TOP2A,TPM1,ZWINT | |
